# Supplementary material for: The cell surface protein MUL_3720 confers binding of the skin pathogen Mycobacterium ulcerans to sulfated glycans and keratin
Source: PLoS Negl Trop Dis. 2021 Feb 25;15(2):e0009136. doi: 10.1371/journal.pntd.0009136 (PMC7906334; doi:10.1371/journal.pntd.0009136)
Supplement: S1 Data — Binding is determined by positive interaction in three replicate array experiments. Positive interactions are determined by a background subtracted fluorescence value significantly above background subtracted fluorescence of negative control spots (average background fluorescence from 20 spots + 3 standard deviations). (PDF) [file pntd.0009136.s001.pdf]

Number	Structure	*M. Ulcerans*	MUL_3720
	**MONOSACCHARIDES**	0.920787555	0.476010763
1	Fucα-sp3	0.747180059	0.442170551
2	Galα-sp3	0.872929149	0.449034929
3	Galβ-sp3	-0.199500072	0.406895425
4	GalNAcα-sp0	1.48±0.63	0.392031872
5	GalNAcα-sp3	0.776200004	0.440481394
6	GalNAcβ-sp3	0.859637051	0.386346864
7	Glcα-sp3	0.874096112	0.472250321
9	Glcβ-sp3	1.63±0.71	0.45786081
10	GlcNAcβ-sp3	-0.322569433	0.484333845
14	GlcN(Gc)β-sp4	0.879256231	0.451183075
15	$HOCH_2(HOCH)_4CH_2NH_2$	0.883151461	0.298274408
16	Manα-sp3	0.941924387	0.51183478
18	Manβ-sp4	0.924517528	0.471586583
19	ManNAcβ-sp4	1.72±0.45	0.357542812
20	Rhaα-sp3	0.734226558	0.044822626
22	GlcNAcβ-sp4	-1.087420131	0.478857457
37	3-O-Su-Galβ-sp3	0.899241149	0.460953294
38	3-O-Su-GalNAcα-sp3	0.644240559	0.477314625
43	6-O-Su-GlcNAcβ-sp3	0.87211583	0.473805647
44	GlcAα-sp3	0.751235473	0.407038978
45	GlcAβ-sp3	0.830702228	0.357495569
46	6-H_2PO_3Glcβ-sp4	0.976710674	0.454427977
47	6-H_2PO_3Manα-sp3	0.207985162	0.426800716
55	3-O-Su-GlcNAcβ-sp3	0.977493738	0.457880157
18G	6-O-Su-GlcNAc	1.30±0.675591	0.44158139
18H	GlcNAc	0.663280187	0.423743282
18I	GlcA	0.898280122	0.496546745
18J	6-O-(H_2PO_4)-Glc	1.40±0.88	0.480740668
	**Terminal Galactose**		
75	Galα1-2Galβ-sp3	0.673724589	0.489772611
76	Galα1-3Galβ-sp3	0.888892461	0.459561037
77	Galα1-3GalNAcβ-sp3	0.919641139	0.474775504
78	Galα1-3GalNAcα-sp3	0.807138197	0.448447744
80	Galα1-3GlcNAcβ-sp3	0.877806152	0.444873471
81	Galα1-4GlcNAcβ-sp3	0.803672498	0.453382636
83	Galα1-6Glcβ-sp4	0.929070952	0.460354819
84	Galβ1-2Galβ-sp3	0.804320445	0.407600325
85	Galβ1-3GlcNaAcβ-sp3	0.961221094	0.401341457
87	Galβ1-3Galβ-sp3	0.845210693	0.474209982
88	Galβ1-3GalNAcβ-sp3	0.86853652	0.520746636
89	Galβ1-3GalNAcα-sp3	0.995935627	0.548875112
93	Galβ1-4Glcβ-sp4	0.969536261	0.466712215
94	Galβ1-4Galβ-sp4	0.952538601	0.501675612
97	Galβ1-4GlcNAcβ-sp3	0.92900512	0.488983882
100	Galβ1-6Galβ-sp4	0.940477463	0.411595595
145	Galβ1-3(6-O-Su)GlcNAcβ-sp3	1.92±0.64	0.432098664
146	Galβ1-4(6-O-Su)Glcβ-sp2	1.59±0.18	0.382382536
147	Galβ1-4(6-O-Su)GlcNAcβ-sp3	1.94±0.34	0.441459242
150	3-O-Su-Galβ1-3GalNAcα-sp3	0.925034296	0.484674178
151	6-O-Su-Galβ1-3GalNAcα-sp3	1.87±0.40	2.44±0.88
152	3-O-Su-Galβ1-4Glcβ-sp2	1.91±0.98	0.432045749
153	6-O-Su-Galβ1-4Glcβ-sp2	0.835687161	0.387696048
155	3-O-Su-Galβ1-3GlcNAcβ-sp3	0.784213452	0.386995928
157	3-O-Su-Galβ1-4GlcNAcβ-sp3	0.948129348	0.458779985
159	4-O-Su-Galβ1-4GlcNAcβ-sp3	1.73±0.26	0.234258854
161	6-O-Su-Galβ1-3GlcNAcβ-sp3	1.64±0.37	2.49±0.26
163	6-O-Su-Galβ1-4GlcNAcβ-sp3	0.883445625	0.456559203
176	3-O-Su-Galβ1-4(6-O-Su)Glcβ-sp2	0.868553257	0.45355879
177	3-O-Su-Galβ1-4(6-O-Su)GlcNAcβ-sp2	0.859341723	0.489597911
178	6-O-Su-Galβ1-4(6-O-Su)Glcβ-sp2	1.17±0.26	0.442476024
179	6-O-Su-Galβ1-3(6-O-Su)GlcNAcβ-sp2	1.60±0.41	1.48±0.33
180	6-O-Su-Galβ1-4(6-O-Su)GlcNAcβ-sp2	1.83±0.51	0.456808464
181	3,4-O-Su_2-Galβ1-4GlcNAcβ-sp3	0.88875458	0.473354359

182	3,6-O-Su$_2$-Galβ1-4GlcNAcβ-sp2	1.99±0.62	3.29±1.77
183	4,6-O-Su$_2$-Galβ1-4GlcNAcβ-sp2	0.861291846	0.482344101
184	4,6-O-Su$_2$-Galβ1-4GlcNAcβ-sp3	0.915892962	0.449957721
189	3,6-O-Su$_2$-Galβ1-4(6-O-Su)GlcNAcβ-sp2	1.90±0.45	2.46±0.56
201	3,4-O-Su$_2$-Galβ1-4GlcNAcβ-sp3	1.92±0.92	1.52±0.64
203	Galβ1-4(6-O-Su)GlcNAcβ-sp2	1.92±0.79	0.512238745
220	Galα1-3Galβ1-4Glcβ-sp2	0.874219413	0.457048289
222	Galα1-3Galβ1-4GlcNAcβ-sp3	0.757108235	0.479131186
224	Galα1-4Galβ1-4Glcβ-sp3	0.884395978	0.501872371
225	Galα1-4Galβ1-4GlcNAc-sp2	0.825614127	0.453322862
228	Galβ1-2Galα1-4GlcNAcβ-sp4	0.319780286	0.518549861
229	Galβ1-3Galβ1-4GlcNAcβ-sp4	0.905304348	0.481293767
231	Galβ1-4GlcNAcβ1-3GalNAcα-sp3	0.854609453	0.402476669
232	Galβ1-4GlcNAcβ1-6GalNAcα-sp3	0.884390562	0.424883793
254	Galβ1-3(GlcNAcβ1-6)GalNAcα-sp3	0.757818506	0.188654539
262	Galβ1-3GalNAcβ1-3Gal-sp4	0.726861231	0.451441538
264	Galβ1-4Galβ1-4GlcNAc-sp3	0.83713815	0.409926491
373	Galα1-3Galβ1-4GlcNAcβ1-3Galβ-sp3	0.811106066	0.435391259
375	Galα1-4GlcNAcβ1-3Galβ1-4GlcNAcβ-sp3	0.832155425	0.079391131
376	Galβ1-3GlcNAcβ1-3Galβ1-4Glcβ-sp4	0.656812183	0.235050814
377	Galβ1-3GlcNAcβ1-3Galβ1-3GlcNAcβ-sp2	0.827730811	0.368109065
378	Galβ1-3GlcNAcα1-3Galβ1-4GlcNAcβ-sp3	0.525654527	0.448976947
379	Galβ1-3GlcNAcβ1-3Galβ1-4GlcNAcβ-sp3	0.881886672	0.424480539
380	Galβ1-3GlcNAcα1-6Galβ1-4GlcNAcβ-sp2	0.818916978	0.463891493
381	Galβ1-3GlcNAcβ1-6Galβ1-4GlcNAcβ-sp2	0.862884901	0.476556224
382	Galβ1-3GalNAcβ1-4Galβ1-4Glcβ-sp3	0.90404305	0.12614185
383	Galβ1-4GlcNAcβ1-3Galβ1-4Glcβ-sp2	0.768682206	0.412037364
385	Galβ1-4GlcNAcβ1-3Galβ1-4GlcNAcβ-sp3	0.796139716	0.339512379
387	Galβ1-4GlcNAcβ1-6Galβ1-4GlcNAcβ-sp2	0.627581702	0.422173635
388	Galβ1-4GlcNAcβ1-6(Galβ1-3)GalNAcα-sp3	0.568127489	0.34006898
488	Galβ1-4GlcNAcβ1-3(Galβ1-4GlcNAcβ1-6)GalNAcα-sp3	0.761925577	0.467361713
489	Galβ1-4GlcNAcβ1-3(GlcNAcβ1-6)Galβ1-4GlcNAc-sp2	0.545387456	0.500069269
501	Galβ1-3GalNAcβ1-3Galα1-4Galβ1-4Glcβ-sp4	0.889001283	0.395774498
504	(A-GN-M)$_2$-3,6-M-GN-GNβ-sp4	0.831443239	0.409804815
1A	Galβ1-3Glc*N*Ac	0.937335379	0.339514143
1B	Galβ1-4Glc*N*Ac	0.804732298	0.0170276
1C	Galβ1-4Gal	0.19309763	0.194282576
1D	Galβ1-6Glc*N*Ac	0.04733912	0.451588212
1E	Galβ1-3Gal*N*Ac	0.886346332	0.37004195
1F	Galβ1-3GalNAcβ1-4Galβ1-4Glc	0.430171248	0.291347651
1G	Galβ1-3Glc*N*Acβ1-3Galβ1-4Glc	0.820709494	0.456665213
1H	Galβ1-4Glc*N*Acβ1-3Galβ1-4Glc	0.915429829	0.480956419
1I	Galβ1-4Glc*N*Acβ1-6(Galβ1-4Glc*N*Acβ1-3)Galβ1-4Glc	0.843813174	0.422841272
1J	Galβ1-4Glc*N*Acβ1-6(Galβ1-3Glc*N*Acβ1-3)Galβ1-4Glc	0.9092585	0.459023362
1K	Gala1-4Galβ1-4Glc	0.895222588	0.463113015
1L	Gal*N*Acα1-*O*-Ser	0.628155913	0.499345819
1M	Galb1-3Gal*N*Acα1-*O*-Ser	0.429982275	0.405683259
1N	Gala1-3Gal	0.817711907	0.250528429
1O	Gala1-3Galβ1-4Glc*N*Ac	0.707202866	0.455061113
1P	Gala1-3Galβ1-4Glc	0.831520628	0.389798106
2A	Gala1-3Galβ1-4Galα1-3Gal	0.766325559	0.437227645
2B	Galβ1-6Gal	0.694973127	0.451248187
2E	Galα1-4Galβ1-4Glc*N*Ac	0.894591519	0.490288431
2G	Galβ1-3Glc*N*Acβ1-3Galβ1-4Glc*N*Acβ1-6(Galβ1-3Glc*N*Acβ1-3)Galβ1-4Glc	0.838244146	0.46756911
2H	Galβ1-3Glc*N*Acβ1-3Galβ1-4Glc*N*Acβ1-3Galβ1-4Glc	0.899102017	0.413832231
18B	Galβ1-3GalNAcβ1-3Galα1-4Galβ1-4Glc	0.793790977	0.456403674
18C	Galβ1-3GalNAcβ1-3Gal	1.77±0.94	0.488285019
18L	Galβ1-4Glc	0.813530545	0.466459096
18M	Galβ1-4Gal	0.841419275	0.329809559
18N	Galβ1-6Gal	0.6304013	0.254620019
19A	Galβ1-4GlcNAcβ1-2Manα1-3(Galβ1-4GlcNAcβ1-2Manα1-6Man)β1-4GlcNAcβ1-4(Fucα1-6)GlcNAc	0.605365828	0.460606982
19B	Galβ1-4GlcNAcβ1-2(Galβ1-4GlcNAcβ1-4)Manα1-3(Galβ1-4GlcNAcβ1-2(Galβ1-4GlcNAcβ1-6)Manα1-6Man)β1-4GlcNAcβ1-4GlcNAc	0.896839927	0.143873204
	**Terminal N-Acetylgalactosamine**		
101	GalNAcα1-3GalNAcβ-sp3	0.955004469	0.302231428
102	GalNAcα1-3Galβ-sp3	0.866848861	0.488714013

103	GalNAcα1-3GalNAcα-sp3	0.670244651	0.471804395
104	GalNAcβ1-3Galβ-sp3	0.95593553	0.452364561
106	GalNAcβ1-4GlcNAcβ-sp3	0.646382379	0.262193126
192	GalNAcβ1-4(6-O-Su)GlcNAcβ-sp3	0.728394656	0.442724247
193	3-O-Su-GalNAcβ1-4GlcNAcβ-sp3	1.52±0.95	0.261000736
194	6-O-Su-GalNAcβ1-4GlcNAcβ-sp3	0.765836967	0.45248252
195	6-O-Su-GalNAcβ1-4-(3-O-Su)GlcNAcβ-sp3	1.93±0.86	0.409689342
196	3-O-Su-GalNAcβ1-4(3-O-Su)-GlcNAcβ-sp3	0.799635554	0.433089083
197	3,6-O-Su_2-GalNAcβ1-4GlcNAcβ-sp3	0.728182996	0.454664967
198	4,6-O-Su_2-GalNAcβ1-4GlcNAcβ-sp3	0.550784193	0.185031325
199	4,6-O-Su_2-GalNAcβ1-4-(3-O-Ac)GlcNAcβ-sp3	1.54±0.79	2.45±0.87
200	4-O-Su-GalNAcβ1-4GlcNAcβ-sp3	0.835119939	0.375418584
202	6-O-Su-GalNAcβ1-4(6-O-Su)GlcNAcβ-sp3	-0.062964585	0.492112265
204	4-O-Su-GalNAcβ1-4GlcNAcβ-sp2	0.970430337	0.40487193
238	GalNAcβ1-4Galβ1-4Glcβ-sp3	0.82623681	0.377654369
389	GalNAcβ1-3Galα1-4Galβ1-4Glcβ-sp3	0.814235161	0.137708606
1L	Gal*N*Acα1-*O*-Ser	0.958391643	0.362882972
2C	Gal*N*Acβ1-3Gal	1.76±0.40	0.414296222
2D	Gal*N*Acβ1-4Gal	0.933598561	0.381359003
2F	GalNAcα1-3Galβ1-4Glc	1.89±0.34	0.557755671
	**Fucosylated**		
71	Fucα1-2Galβ-sp3	0.167097516	0.451908743
72	Fucα1-3GlcNAcβ-sp3	0.929376404	0.484350149
73	Fucα1-4GlcNAcβ-sp3	0.799830886	0.495833447
215	Fucα1-2Galβ1-3GlcNAcβ-sp3	1.86±0.26	0.361827137
216	Fucα1-2Galβ1-4GlcNAcβ-sp3	0.575272448	0.506331733
217	Fucα1-2Galβ1-3GalNAcα-sp3	0.895498052	0.501775833
219	Fucα1-2Galβ1-4Glcβ-sp4	1..88±0.93	0.420709849
226	Fucα1-2(Galα1-3)Galβ-sp3	0.916524746	0.45471304
233	Galβ1-3(Fucα1-4)GlcNAcβ-sp3	0.748948306	0.467525887
234	Fucα1-3(Galβ1-4)GlcNAcβ-sp3	0.813291448	0.420609356
235	Fucα1-2(GalNAcα1-3)Galβ-sp3	0.741994439	0.463053117
287	3-O-Su-Galβ1-3(Fucα1-4)GlcNAcβ-sp3	0.92517507	0.472921708
288	Fucα1-3(3-O-Su-Galβ1-4)GlcNAcβ-sp3	0.609906675	0.488055561
359	Fucα1-2(Galα1-3)Galβ1-3GlcNAcβ-sp3	0.699535171	0.471606928
360	Fucα1-2(Galα1-3)Galβ1-4GlcNAcβ-sp3	-0.245381843	0.13677492
362	Fucα1-2(Galα1-3)Galβ1-3GalNAcα-sp3	0.805766151	0.530175256
363	Fucα1-2(Galα1-3)Galβ1-3GalNAcβ-sp3	0.639705964	0.488612425
364	Fucα1-3(Galα1-3Galβ1-4)GlcNAcβ-sp3	0.741565036	0.524830825
366	Fucα1-2(GalNAcα1-3)Galβ1-3GlcNAcβ-sp3	-0.682435477	0.427363963
368	Fucα1-2(GalNAcα1-3)Galβ1-4GlcNAcβ-sp3	-0.059796745	0.473387026
371	Fucα1-2Galβ1-3(Fucα1-4)GlcNAcβ-sp3	0.472436259	0.495551198
372	Fucα1-3(Fucα1-2Galβ1-4)GlcNAcβ-sp3	0.795907789	0.510308692
392	Fucα1-2(GalNAcα1-3)Galβ1-3GalNAcα-sp3	0.697922155	0.179187755
479	Fucα1-2Galβ1-3GlcNAcβ1-3Galβ1-4Glcβ-sp4	0.855565974	0.502401819
480	Fucα1-2Galβ1-3GlcNAcβ1-3Galβ1-4GlcNAcβ-sp2	0.906224895	0.488722336
483	Fucα1-3(Fucα1-2 (Galα1-3)Galβ1-4)GlcNAcβ-sp3	-0.4954326	0.484558609
496	Fucα1-2Galβ1-3(Fucα1-4)GlcNAcβ1-3Galβ1-4Glcβ-sp4	0.648149457	0.249479556
497	Fucα1-3(Fucα1-2Galβ1-4)GlcNAcβ1-3Galβ1-4Glcβ-sp4	0.358049515	0.445799172
538	Le^x1-6'(Le^c1-3')Lac-sp4	0.688694538	0.307534896
539	LacNAc1-6'(Le^d1-3')Lac-sp4	0.360275918	0.402524923
541	Le^x1-6'(Le^d1-3')Lac-sp4	0.869446852	0.407979451
542	Le^cLe^x1-6'(Le^c1-3')Lac-sp4	2.77±0.76	0.045968782
543	Le^x1-6'(Le^b1-3')Lac-sp4	0.583097992	0.477575527
7A	Fucα1-2Galβ1-3Glc*N*Acβ1-3Galβ1-4Glc	1.63±0.92	0.325578581
7B	Galβ1-3(Fuca1-4)Glc*N*Acβ1-3Galβ1-4Glc	0.358056573	0.194268627
7C	Galβ1-4(Fuca1-3)Glc*N*Acβ1-3Galβ1-4Glc	-0.931889598	0.419817557
7D	Fucα1-2Galβ1-3(Fucα1-4)Glc*N*Acβ1-3Galβ1-4Glc	0.082986544	0.403067987
7E	Galβ1-3(Fucα1-4)Glc*N*Acβ1-3Galβ1-4(Fucα1-3)Glc	1.82±0.72	0.459276732
7F	Fucα1-2Gal	0.29865439	0.223128005
7G	Fucα1-2Galβ1-4Glc	1.69±0.59	0.444366978
7H	Galβ1-4(Fucα1-3)Glc	0.831317794	0.279819522
7I	Galβ1-4(Fucα1-3)Glc*N*Ac	0.803860284	0.276971755
7J	Galβ1-3(Fucα1-4)Glc*N*Ac	0.972418298	0.418564276
7K	Gal*N*Acα1-3(Fucα1-2)Gal	0.318725109	0.382111501

7L	Fucα1-2Galβ1-4(Fucα1-3)Glc	1.83±0.80	0.47187091
7M	Galβ1-3(Fucα1-2)Gal	0.546125437	0.347017434
7N	Fucα1-2Galβ1-4(Fucα1-3)Glc*N*Ac	0.710998717	0.352614801
7O	Fucα1-2Galβ1-3Glc*N*Ac	0.768556761	0.22684152
7P	Fucα1-2Galβ1-3(Fucα1-4)Glc*N*Ac	1.66±0.26	0.473628573
8A	SO_3-3Galβ1-3(Fucα1-4)Glc*N*Ac	0.795267702	0.169514408
8B	SO_3-3Galβ1-4(Fucα1-3)Glc*N*Ac	0.40690237	0.499979433
8C	Galβ1-3Glc*N*Acβ1-3Galβ1-4(Fucα1-3)Glc*N*Acβ1-3Galβ1-4Glc	0.55266088	0.098436117
8D	Galβ1-4(Fucα1-3)Glc*N*Acβ1-6(Galβ1-3Glc*N*Acβ1-3)Galβ1-4Glc	0.713653668	0.427361296
8E	Galβ1-4(Fucα1-3)Glc*N*Acβ1-6(Fucα1-2Galβ1-3Glc*N*Acβ1-3)Galβ1-4Glc	0.169828752	0.233102359
8F	Galβ1-4(Fucα1-3)Glc*N*Acβ1-6(Fucα1-2Galβ1-3(Fucα1-4)Glc*N*Acβ1-3)Galβ1-4Glc	0.420709494	0.481238077
8G	Galβ1-4GlcNAcβ1-3Galβ1-4(Fucα1-3)Glc	0.684570171	0.434408711
8H	Fucα1-2Galβ1-4(Fucα1-3)GlcNAcβ1-3Galβ1-4Glc	0.756186826	0.378710743
8I	Fucα1-3Galβ1-4GlcNAcβ1-3Galβ1-4(Fucα1-3)Glc	0.6907415	0.421651975
8J	Fuca1-2Galb1-4(Fuca1-3)GlcNAcb1-3(Fuca1-2)Galb1-4Glc	0.704777412	0.458439023
8K	Galβ1-4(Fucα1-3)GlcNAcβ1-6(Galβ1-4GlcNAcβ1-3)Galβ1-4Glc	0.971844087	0.476043452
8L	Galb1-4(Fuca1-3)GlcNAcb1-6(Galb1-4(Fuca1-3)GlcNAcb1-3)Galb1-4Glc	0.170017725	0.460067223
8M	Fuca1-2Galb1-4(Fuca1-3)GlcNAcb1-6(Galb1-4GlcNAcb1-3)Galb1-4Glc	0.782288093	0.387126818
8N	Galb1-3GlcNAcb1-3Galb1-4(Fuca1-3)GlcNAcb1-6(Galb1-3GlcNAcb1-3)Galb1-4Glc	0.892797134	0.459868042
8O	Fucα1-2Galβ1-3GlcNAcβ1-3Galβb1-4(Fucα1-3)GlcNAcβ1-6(Galβ1-3GlcNAcβ1-3)Galβ1-4Glc	0.768479372	0.441916779
8P	GalNAcb1-3(Fuca1-2)Galb1-4Glc	0.833674441	0.47043936
9A	Galb1-3(Fuca1-2)Galb1-4(Fuca1-3)Glc	0.905026873	0.415918983
9B	Galβ1-4Glc*N*Acβ1-6(Fucα1-2Galβ1-3Glc*N*Acβ1-3)Galβ1-4Glc	0.613813019	0.439381805
18D	Galα1-3(Fucα1-2)Galβ1-4Glc	0.677037329	0.438175354
18E	GalNAcα1-3(Fucα1-2)Galβ1-4(Fucα1-3)Glc	0.381054085	0.435196561
19J	Galβ1-4(Fucα1-3)GlcNAcβ1-3Gal	0.640909555	0.433552898
19L	Fucα1-2Galβ1-4(Fucα1-3)GlcNAcβ1-3Gal	0.761755854	0.45342365
19M	Galβ1-3(Fucα1-4)GlcNAcβ1-3Gal	0.700897983	0.434937707
19N	Fucα1-2Galβ1-3(Fucα1-4)GlcNAcβ1-3Gal	0.806209023	0.449707839
	**Neu5Ac**		
48	Neu5Acα-sp3	0.786469455	0.457675557
49	Neu5Acα-sp9	0.758580725	0.494177668
54	9-NAc-Neu5Acα-sp3	0.9695987	0.301996291
18K	9-NAc-Neu5Ac	1.99±0.46	0.494373435
169	Neu5Acα2-3Galβ-sp3	1.70±0.31	0.327168898
171	Neu5Acα2-3GalNAcα-sp3	1.51±0.70	0.415820047
292	Neu5Acα2-3Galβ1-3GalNAcα-sp3	0.404499553	0.474570031
293	Neu5Acα2-3Galβ1-4Glcβ-sp3	0.733151139	0.485220207
294	Neu5Acα2-3Galβ1-4Glcβ-sp4	0.593297553	0.465721225
315	Neu5Acα2-3Galβ1-4-(6-O-Su)GlcNAcβ-sp3	0.64406447	0.418431147
317	Neu5Acα2-3Galβ1-3-(6-O-Su)GalNAcβ-sp3	0.953617621	0.472223115
318	Neu5Acα2-6Galβ1-4-(6-O-Su)GlcNAcβ-sp3	0.871605344	0.479910285
319	Neu5Acα2-3-(6-O-Su)Galβ1-4GlcNAcβ-sp3	0.070469857	0.451784549
421	Neu5Acα2-3(GalNAcβ1-4)Galβ1-4Glcβ-sp2	1.83±0.41	0.469451538
422	Neu5Acα2-3Galβ1-4GlcNAcβ1-3Galβ-sp3	0.661303286	0.513450918
423	Fucα1-3(Neu5Acα2-3Galβ1-4)GlcNAcβ-sp3	0.800364446	0.482267738
426	Neu5Acα2-3Galβ1-3(Fucα1-4)GlcNAcβ-sp3	0.871817004	0.514510801
428	Fucα1-3(Neu5Acα2-3Galβ1-4)6-O-Su-GlcNAcβ-sp3	0.049215807	0.519740305
429	Fucα1-3(Neu5Acα2-3(6-O-Su)Galβ1-4)GlcNAcβ-sp3	0.052016289	0.461302673
433	Neu5Acα2-3Galβ1-3(Neu5Acα2-6)GalNAcα-sp3	0.764880061	0.503031467
527	Neu5Acα2-3Galβ1-4GlcNAcβ1-3Galβ1-4GlcNAcβ-sp2	0.662964585	0.498983907
528	Fucα1-3(Neu5Acα2-3 Galβ1-4)GlcNAcβ1-3Galβ-sp3	0.629569663	0.492384065
534	Neu5Acα2-3Galβ1-4GlcNAcβ1-3Galβ1-4GlcNAcβ-sp3	0.77376319	0.48813465
536	Neu5Acα2-3Galβ1-3GlcNAcβ1-3Galβ1-4Glcβ-sp4	0.785764839	0.48225128
537	Neu5Acα2-3Galβ1-4GlcNAcβ1-3Galβ1-4Glcβ-sp4	0.641608357	0.505985119
10A	Neu5Acα2-3Galβ1-3(Fucα1-4)Glc*N*Ac	0.830095904	0.481016113
10B	Neu5Acα2-3Galβ1-4(Fucα1-3)Glc*N*Ac	0.666401439	0.399545792
10C	Neu5Acα2-3Galβ1-3Glc*N*Acβ1-3Galβ1-4Glc	0.772766144	0.487336628
10E	Neu5Acα2-3Galβ1-3(Neu5Acα2-6)GalNAc	0.706582563	0.481258574
10K	Neu5Acα2-3Galβ1-4Glc*N*Ac	0.432902484	0.469510147
10M	Neu5Acα2-3Galβ1-3GlcNAcβ1-3Galβ1-4Glc	0.670623596	0.477745362
10P	Neu5Acα2-3Galβ1-3(Neu5Acα2-6)Glc*N*Acβ1-3Galβ1-4Glc	0.800169114	0.45892179
11A	Neu5Acα2-3Galβ1-4Glc	0.737308778	0.519605336
18A	Neu5Acα2-3Galβ1-4GlcNAcβ1-3Galβ1-4Glc	0.494247276	0.487032337
19K	Neu5Acα2-3Galβ1-4(Fucα1-3)GlcNAcβ1-3Gal	0.551045019	0.503668315

170	Neu5Acα2-6Galβ-sp3	1.77±0.18	0.51609463
172	Neu5Acα2-6GalNAcα-sp3	1.68±0.34	0.470861406
205	Neu5Acα2-6GalNAcβ-sp3	0.592510517	0.467138314
289	Galα1-3(Neu5Acα2-6)GalNAcα-sp3	0.786708552	0.526214835
290	Galβ1-3(Neu5Acα2-6) GalNAcα-sp3	0.525800556	0.441204392
295	Neu5Acα2-6Galβ1-4Glcβ-sp2	0.67482493	0.509010494
298	Neu5Acα2-3Galβ1-4GlcNAcβ-sp3	0.990093325	0.458791579
299	Neu5Acα2-3Galβ1-3GlcNAcβ-sp3	0.900464829	0.472188645
300	Neu5Acα2-6Galβ1-4GlcNAcβ-sp3	0.845381843	0.499052261
306	9-NAc-Neu5Acα2-6Galβ1-4GlcNAcβ-sp3	0.794233849	0.500621532
323	Neu5Acα2-6Galβ1-3GlcNAc-sp3	0.960294036	0.522897324
324	Neu5Acα2-6Galβ1-3(6-O-Su)GlcNAc-sp3	0.858434964	0.476012986
529	Neu5Acα2-6(Galβ1-3)GlcNAcβ1-3Galβ1-4Glcβ-sp4	0.282435477	0.481090231
540	Le^{x}1-6'(6'SLN1-3')Lac-sp4	0.659796745	0.529685549
10D	Galβ1-4(Fucα1-3)Glc*N*Acβ1-6(Neu5Acα2-6Galβ1-4Glc*N*Acβ1-3)Galβ1-4Glc	0.127563741	0.480198158
10H	Neu5Acα2-6Galβ1-3GlcNAcβ1-3Galβ1-4(Fucα1-3)Glc	0.804092211	0.504392771
10I	Galβ1-3GlcNAcβ1-3(Neu5Acα2-6Galβ1-4GlcNAcβ1-6)Galβ1-4Glc	0.902077845	0.471098994
10J	Neu5Acα2-6Galβ1-3GlcNAcβ1-3(Galβ1-4GlcNAcβ1-6)Galβ1-4Glc	0.744434026	0.456403532
10L	Neu5Acα2-6Galβ1-4Glc*N*Ac	0.693775105	0.329945071
10N	Galβ1-3(Neu5Acα2-6)Glc*N*Acβ1-3Galβ1-4Glc	0.0954326	0.476326087
10O	Neu5Acα2-6Galβ1-4Glc*N*Acβ1-3Galβ1-4Glc	0.951850543	0.463652363
11B	Neu5Acα2-6Galβ1-4Glc	0.241950485	0.471097641
11D	Neu5Acα2-6Galβ1-4GlcNAcβ1-2Manα1-6(Neu5Acα2-6Galβ1-4GlcNAcβ1-2Manα1-6)Manβ1-4GlcNAcβ1-4GlcNAc-Asn	0.911305462	0.439454627
627	(Sia2-6A-GN-M)$_2$-3,6-M-GN-GNβ-sp4	0.239724082	0.431715308
19C	Neu5Acα2-6Galβ1-4GlcNAcβ1-2Manα1-3(Galβ1-4GlcNAcβ1-2Manα1-6)Manβ1-4GlcNAcβ1-4GlcNAc	0.730553148	0.459284537
19D	Neu5Acα2-6Galβ1-4GlcNAcβ1-2Manα1-3(Neu5Acα2-6Galβ1-4GlcNAcβ1-2Manα1-6)Manβ1-4GlcNAcβ1-4GlcNAc	0.599722924	0.452776517
19E	Galβ1-4GlcNAcβ1-2Manα1-3(Galβ1-4GlcNAcβ1-2Manα1-6)Manβ1-4GlcNAcβ1-4GlcNAc	0.016902008	0.458038856
19F	Neu5Acα2-6Galβ1-4GlcNAcβ1-2Manα1-3(Neu5Acα2-6Galβ1-4GlcNAcβ1-2Manα1-6)Manβ1-4GlcNAcβ1-4(Fucα1-6)GlcNAc	0.96078074	0.414203046
19G	Neu5Acα2-6Galβ1-4GlcNAcβ1-2(Neu5Acα2-6Galβ1-4GlcNAcβ1-4)Manα1-3(Neu5Acα2-6Galβ1-4GlcNAcβ1-2Manα1-6)Manβ1-4GlcNAcβ1-4GlcNAc	0.241943427	0.456932703
186	Neu5Acα2-8Neu5Acα2-sp3	0.531889598	0.381413632
321	(Neu5Acα2-8)$_3$-sp3	0.820506117	0.470471668
434	Neu5Acα2-8Neu5Acα2-3Galβ1-4Glcβ-sp4	0.5817486	0.454729245
531	GalNAcβ1-4(Neu5Acα2-8Neu5Acα2-3)Galβ1-4Glc-sp2	0.674226749	0.480196889
532	Neu5Acα2-8Neu5Acα2-8Neu5Acα2-3Galβ1-4Glc-sp2	0.697208322	0.436795015
533	(Neu5Acα2-8)2Neu5Acα2-3(GalNAcβ1-4)Galβ1-4Glc-sp2	1.62±0.77	0.468232287
11C	(Neu5Acα2-8Neu5Ac)n (n<50)	0.661339811	0.200124982
**Neu5Gc**	**Neu5Gc**		
52	Neu5Gcα-sp3	0.694712886	0.444050001
18O	Neu5Gc	1.60±0.59	0.464909263
206	Neu5Gcα2-3Gal-sp3	0.681281276	0.468524028
303	Neu5Gcα2-3Galβ1-4GlcNAcβ-sp3	0.659710337	0.409273653
331	Neu5Gcα2-3Galβ1-3GlcNAcβ-sp3	0.694641578	0.169357642
174	Neu5Gcα2-6GalNAcα-sp3	1.77±0.24	0.469814058
304	Neu5Gcα2-6Galβ1-4GlcNAcβ-sp3	0.718124238	0.470787865
	**Mannose**		
119	Manα1-2Manβ-sp4	1.49±0.14	0.481129382
120	Manα1-3Manβ-sp4	0.676036529	0.431896671
121	Manα1-4Manβ-sp4	0.678081558	0.43355664
122	Manα1-6Manβ-sp4	1.49±0.28	-0.021855033
123	Manβ1-4GlcNAcβ-sp4	1.69±0.54	0.474810287
124	Manα1-2Manα-sp4	0.704854237	0.41106014
258	Manα1-3(Manα1-6)Manβ-sp4	0.663974739	0.468028957
495	Manα1-3(Manα1-3(Manα1-6)Manα1-6)Manβ-sp4	0.671513599	0.437808868
5A	Glc*N*Acβ1-2Man	0.611691604	0.140636036
5B	Glc*N*Acβ1-2Manα1-6(Glc*N*Acβ1-2Manα1-3)Man	0.58560469	0.022746934
5C	Manα1-2Man	0.703454666	0.164727334
5D	Manα1-3Man	1.48±0.34	0.02199973
5E	Manα1-4Man	1.56±0.81	0.213394971
5F	Manα1-6Man	0.670002414	0.223339581
5G	Manα1-6(Manα1-3)Man	0.661056024	0.091372944
5H	Manα1-6(Manα1-3)Manα1-6(Manα1-3)Man	0.674469817	0.055624097
	**Terminal N-Acetylglucosamine**		
113	GlcNAcβ1-3GalNAcα-sp3	0.543798698	0.219819767
114	GlcNAcβ1-3Manβ-sp4	0.645987389	0.098762654
115	GlcNAcβ1-4GlcNAcβ-Asn	0.68105447	0.073901315

117	GlcNAcβ1-4GlcNAcβ-sp4	0.643991712	0.155666128
118	GlcNAcβ1-6GalNAcα-sp3	1.67±0.81	0.107262512
149	GlcNAcβ1-4(6-O-Su)GlcNAcβ-sp2	1.68±0.29	-0.056698508
167	GlcNAcβ1-4-[HOOC(CH_3)CH]-3-O-GlcNAcβ-sp4	0.684862375	0.065898118
168	GlcNAcβ1--[HOOC(CH_3)CH]-3-O-GlcNAcβ-L-alanyl-D-i-glutaminyl-L-lysine	0.725399552	-0.061412218
246	GlcNAcβ1-2Galβ1-3GalNAcα-sp3	0.693229079	-0.085196096
247	GlcNAcβ1-3Galβ1-3GalNAcα-sp3	0.688920462	0.130829578
248	GlcNAcβ1-3Galβ1-4Glcβ-sp2	0.618119711	-0.012272905
250	GlcNAcβ1-3Galβ1-4GlcNAcβ-sp3	0.693134174	0.004047854
251	GlcNAcβ1-4Galβ1-4GlcNAcβ-sp2	0.639468495	0.0295357
252	GlcNAcβ1-4GlcNAcβ1-4GlcNAcβ-sp4	0.419165073	0.045208815
253	GlcNAcβ1-6Galβ1-4GlcNAcβ-sp2	0.693299058	0.065954607
255	GlcNAcβ1-3(GlcNAcβ1-6)GalNAcα-sp3	0.628543061	-0.024513624
395	GlcNAcβ1-3(GlcNAcβ1-6)Galβ1-4GlcNAcβ-sp3	0.654637177	0.070169341
493	$(GlcNAc\beta1\text{-}4)_5\beta$-sp4	1.47±0.32	0.240360066
503	$(GlcNAc\beta1\text{-}4)_6\beta$-sp4	0.589151384	0.048087713
505	$(GN\text{-}M)_2$-3,6-M-GN-GNβ-sp4	0.62353232	0.069345847
4A	Glc*N*Acβ1-4Glc*N*Ac	0.623248663	0.107085313
4B	Glc*N*Acβ1-4Glc*N*Acβ1-4Glc*N*Ac	0.455773278	0.081094248
4C	Glc*N*Acβ1-4Glc*N*Acβ1-4Glc*N*Acβ1-4Glc*N*Ac	1.44±0.59	0.13731413
4D	Glc*N*Acβ1-4Glc*N*Acβ1-4Glc*N*Acβ1-4Glc*N*Acβ1-4Glc*N*Acβ1-4Glc*N*Ac	0.597919938	-0.084571253
4E	Bacterial cell wall muramyl discaccharide	1.48±0.73	0.049180088
4F	Glc*N*Acβ1-4Glc*N*Acβ1-4Glc*N*Acβ1-4Glc*N*Acβ1-4Glc*N*Ac	0.653183605	-0.014888565
19H	GlcNAcβ1-2(GlcNAcβ1-4)Manα1-3(GlcNAcβ1-2Manα1-6)GlcNAcβ1-4Manβ1-4GlcNAcβ1-4GlcNAc	0.641404236	-0.068523113
	**Glucose**		
110	Glcα1-4Glcβ-sp3	0.669720562	0.114168497
111	Glcβ1-4Glcβ-sp4	0.590359785	-0.115854823
112	Glcβ1-6Glcβ-sp4	0.567826047	0.179871656
164	GlcAβ1-3GlcNAcβ-sp3	1.52±0.48	-0.037341002
165	GlcAβ1-3Galβ-sp3	1.45±0.40	0.137663542
166	GlcAβ1-6Galβ-sp3	1.61±0.36	0.098869876
240	$(Glc\alpha1\text{-}4)_3\beta$-sp4	0.522728363	0.003776583
241	$(Glc\alpha1\text{-}6)_3\beta$-sp4	0.642387891	-0.00249231
390	$(Glc\alpha1\text{-}4)_4\beta$-sp4	0.620624027	-0.099977895
391	$(Glc\alpha1\text{-}6)_4\beta$-sp4	0.638424761	0.086742027
492	$(Glc\alpha1\text{-}6)_5\beta$-sp4	0.410879949	0.069917808
502	$(Glc\alpha1\text{-}6)_6\beta$-sp4	0.193690103	-0.132841903
19O	Glcα1-4Glcα1-4	0.249463666	0.072933559
19P	Glcα1-4Glcα1-4Glcα1-4	0.628194141	-0.017879825
	**Low molecular weight Carageenan and Glycoaminoglycans (GAGS)**		
12A	Neocarratetraose-41, 3-di-O-sulphate (Na+)	1.63±0.86	0.14397544
12B	Neocarratetraose-41-O-sulphate (Na+)	1.69±0.81	0.217520855
12C	Neocarrahexaose-24,41, 3, 5-tetra-O-sulphate (Na+)	0.633327223	0.085728386
12D	Neocarrahexaose-41, 3, 5-tri-O-sulphate (Na+)	0.684140931	0.124252136
12E	Neocarraoctaose-41, 3, 5, 7-tetra-O-sulphate (Na+)	1.67±0.91	0.102243896
12F	Neocarradecaose-41, 3, 5, 7, 9-penta-O-sulphate (Na+)	0.563750866	0.183529618
12G	ΔUA-2S-GlcNS-6S	1.41±0.78	0.198533982
12H	ΔUA-GlucNS-6S	0.534120168	0.136337868
12I	ΔUA-2S-GlucNS	0.581131989	0.153213016
12J	ΔUA-2S-GlcNAc-6S	0.610659367	0.139672773
12K	ΔUA-GlcNAc-6S	0.601776602	0.225409939
12L	ΔUA-2S-GlcNAc	0.573110657	0.142592515
12M	ΔUA-GlcNAc	1.73±0.60	0.249341845
12N	ΔUA-GalNAc-4S (Delta Di-4S)	0.708859088	0.10416257
12O	ΔUA-GalNAc-6S (Delta Di-6S)	0.692439975	0.148291961
12P	ΔUA-GalNAc-4S,6S (Delta Di-disE)	0.731738202	0.160711806
13A	ΔUA-2S-GalNAc-4S (Delta Di-disB)	0.652906628	0.164061301
13B	ΔUA-2S-GalNAc-6S (Delta Di-disD)	0.656467124	0.154294359
13C	ΔUA-2S-GalNAc-4S-6S (Delta Di-tisS)	0.625097325	0.156157339
13D	ΔUA-2S-GalNAc-6S (Delta Di-UA2S)	0.633889451	0.141322003
13E	ΔUA-GlcNAc (Delta Di-HA)	0.63999482	0.147237929
14M	ΔUA→2S-GlcN-6S	0.585614417	0.147070123
14N	ΔUA→GlcN-6S	0.44251066	0.144898314
14O	ΔUA→2S-GlcN	0.532986405	0.141847492
14P	ΔUA→GlcN	0.520356566	0.139865902

High molecular weight Carageenan and Glycoaminoglycans (GAGS)

625	(GlcAβ1-4GlcNAcβ1-3)$_8$-NH$_2$-ol	0.628527567	0.806352848
13F	(GlcAβ1-3Glc*N*Acβ1-4)n (n=4)	0.51509245	0.480167799
13G	(GlcAβ1-3Glc*N*Acβ1-4)n (n=8)	0.360759449	0.165390603
13H	(GlcAβ1-3Glc*N*Acβ1-4)n (n=10)	0.102239732	0.826660851
13I	(GlcAβ1-3Glc*N*Acβ1-4)n (n=12)	0.102969453	0.923825675
13J	(GlcA/IdoAα/β1-4Glc*N*Acα1-4)n (n=200)	0.903646795	0.691365087
13K	(GlcA/IdoAβ1-3(±4/6S)Gal*N*Acβ1-4)n (n<250)	2.71±1.74	0.836093449
13L	((±2S)GlcA/IdoAα/b1-3(±4S)Gal*N*Acβ1-4)n (n<250)	2.70±0.58	0.852452061
13M	(GlcA/IdoAβ1-3(±6S)Gal*N*Acβ1-4)n (n<250)	0.962838704	0.997383274
13N	HA - 4 10mM	1.79±0.64	0.622733035
130	HA - 6 10mM	0.91184715	0.002113716
13P	HA - 8 9.7mM	0.879653111	0.82024445
14A	HA 10 7.83mM	0.852452061	0.559192422
14B	HA-12 6.5mM	0.776766828	0.748910579
14C	HA-14 5.6mM	0.779137151	0.804992749
14D	HA-16 4.9mM	0.680110989	0.943583311
14E	HA 30000 da 2.5mg/ml	0.880284203	0.97902202
14F	HA 107000 da 2.5mg/ml	0.910445231	0.961153724
14G	HA 190000 da 2.5 mg/ml	0.920078879	0.795518719
14H	HA 220000 da 2.5 mg/ml	0.895750131	0.833142785
14I	HA 1600000 da 2.5 mg/ml	2.67±0.94	0.846552879
14J	Heparin sulfate 5 mg/ml	0.707398993	0.853169414
14K	β1-3Glucan	0.456980433	0.83877517

Red indicates binding. Binding is determined by positive interaction in three replicate array experiments.
Positive interactions are determined by a background subtracted fluorescence value significantly above background subtracted fluorescence of negative control spots (average background fluorescence from 20 spots + 3 standard deviations).
Values for +ve binding = 2706±372 RFU *M. ulcerans* ; 1829±140 RFU MUL3720 for the three arrays of each sample
