# Supplementary material for: The cell surface protein MUL_3720 confers binding of the skin pathogen Mycobacterium ulcerans to sulfated glycans and keratin
Source: PLoS Negl Trop Dis. 2021 Feb 25;15(2):e0009136. doi: 10.1371/journal.pntd.0009136 (PMC7906334; doi:10.1371/journal.pntd.0009136)
Supplement: S2 Data — (PDF) [file pntd.0009136.s002.pdf]

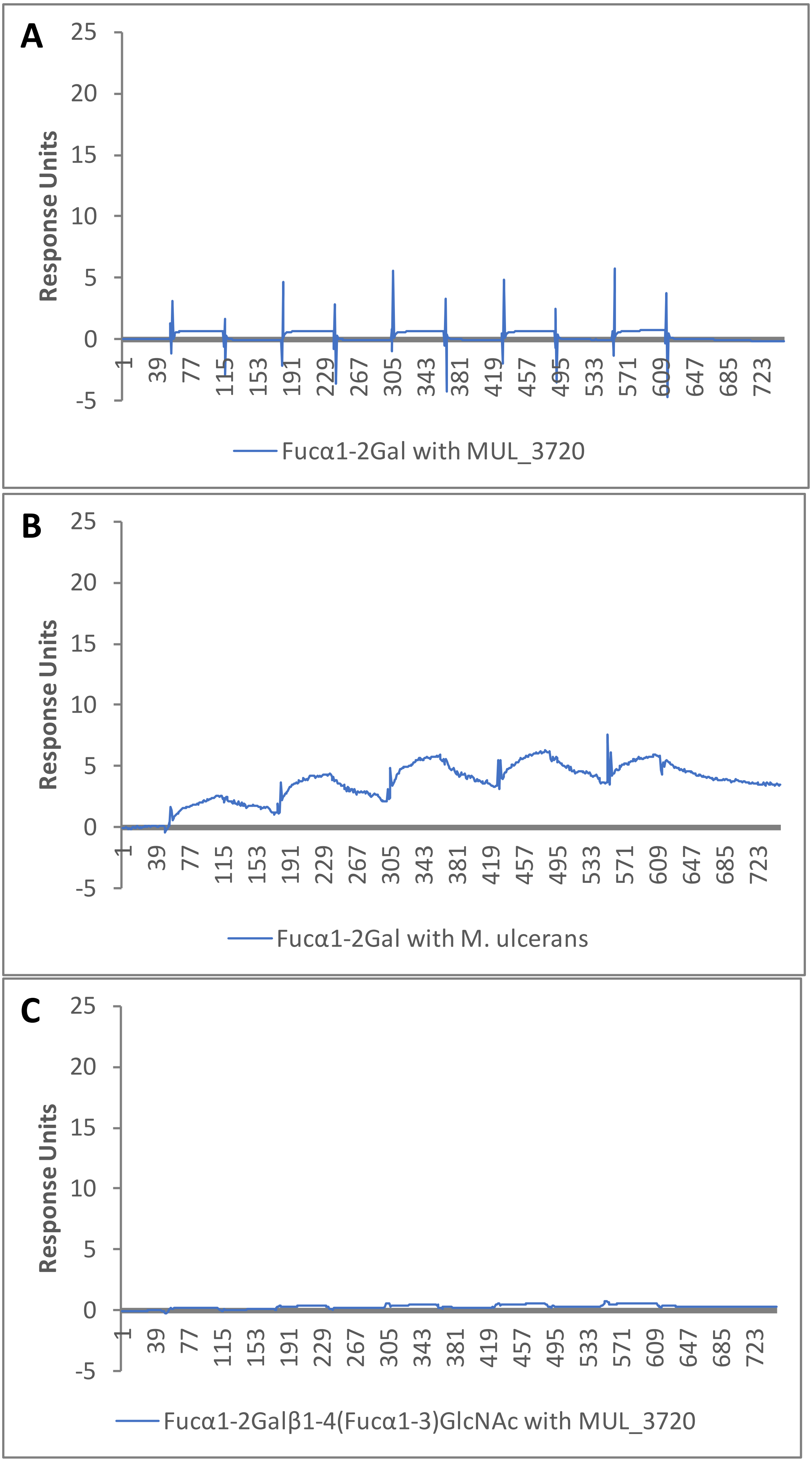
A
Response Units
25
20
15
10
5
0
-5
1 39 77 115 153 191 229 267 305 343 381 419 457 495 533 571 609 647 685 723
Fucα1-2Gal with MUL_3720
B
Response Units
25
20
15
10
5
0
-5
1 39 77 115 153 191 229 267 305 343 381 419 457 495 533 571 609 647 685 723
Fucα1-2Gal with M. ulcerans
C
Response Units
25
20
15
10
5
0
-5
1 39 77 115 153 191 229 267 305 343 381 419 457 495 533 571 609 647 685 723
Fucα1-2Galβ1-4(Fucα1-3)GlcNAc with MUL_3720

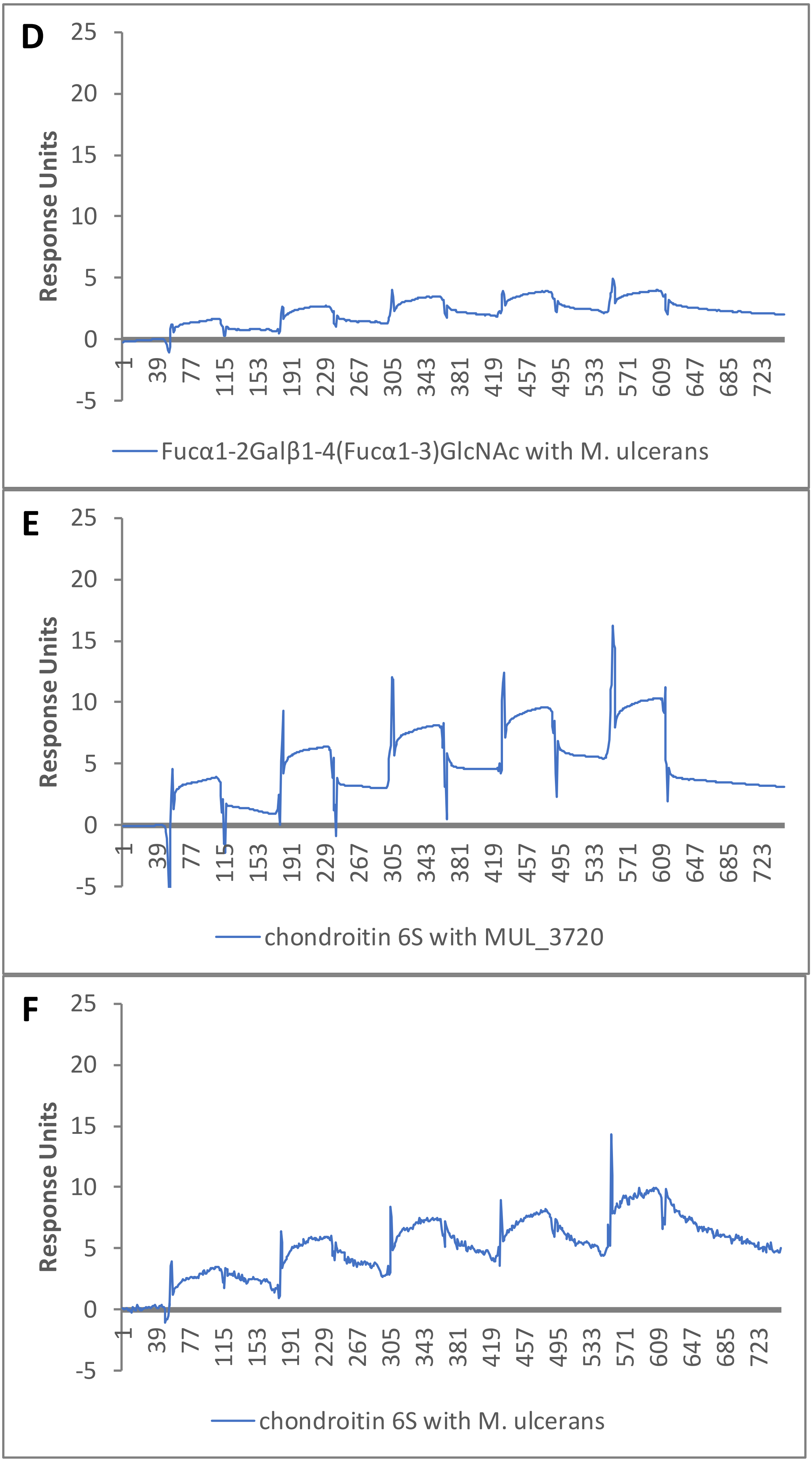
D
25
20
15
10
5
0
-5
Response Units
1
39
77
115
153
191
229
267
305
343
381
419
457
495
533
571
609
647
685
723
Fucα1-2Galβ1-4(Fucα1-3)GlcNAc with M. ulcerans
E
Response Units
chondroitin 6S with MUL_3720
F
Response Units
chondroitin 6S with M. ulcerans

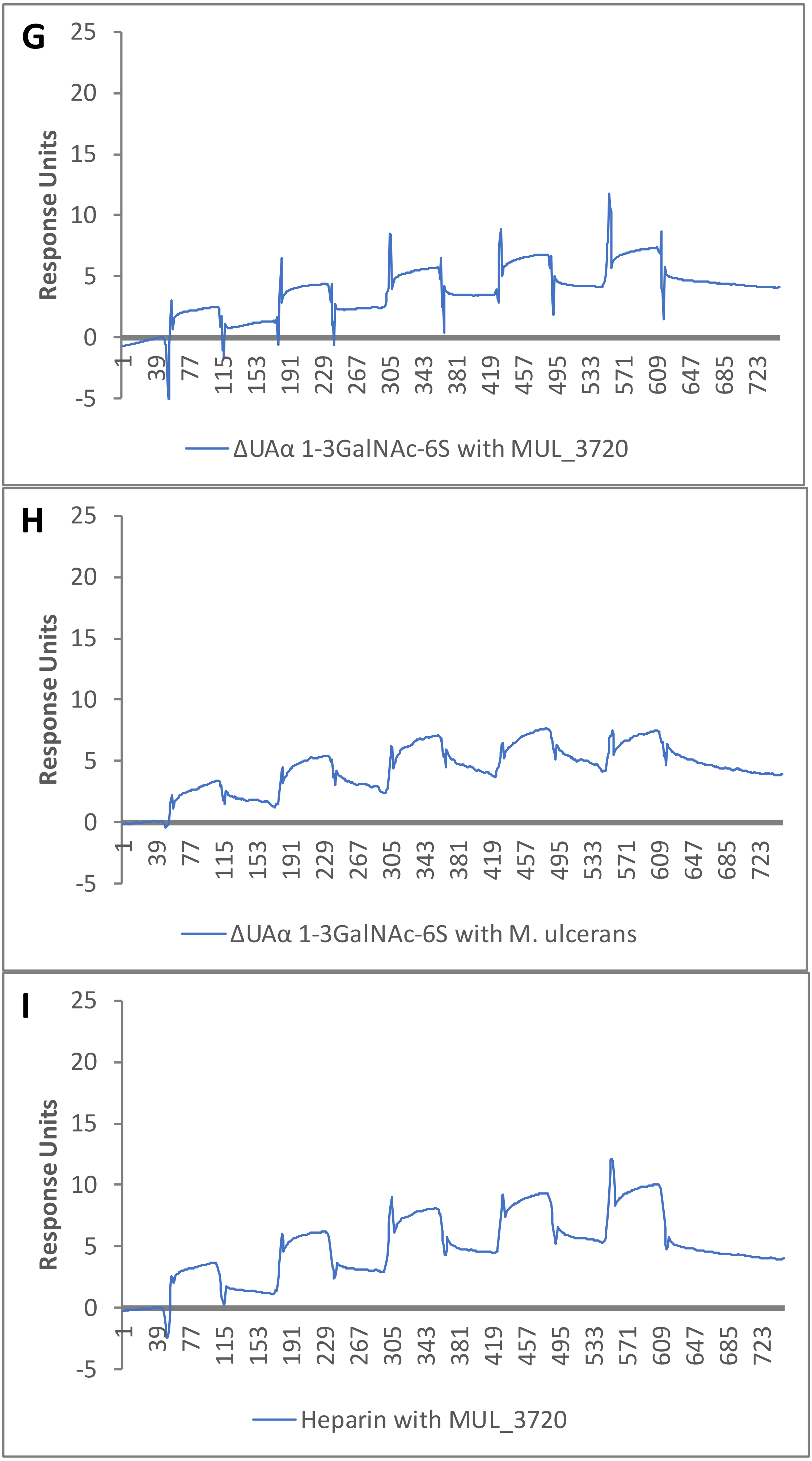
G
Response Units
25
20
15
10
5
0
-5
1
39
77
115
153
191
229
267
305
343
381
419
457
495
533
571
609
647
685
723
ΔUAα 1-3GalNAc-6S with MUL_3720
H
Response Units
ΔUAα 1-3GalNAc-6S with M. ulcerans
I
Response Units
Heparin with MUL_3720

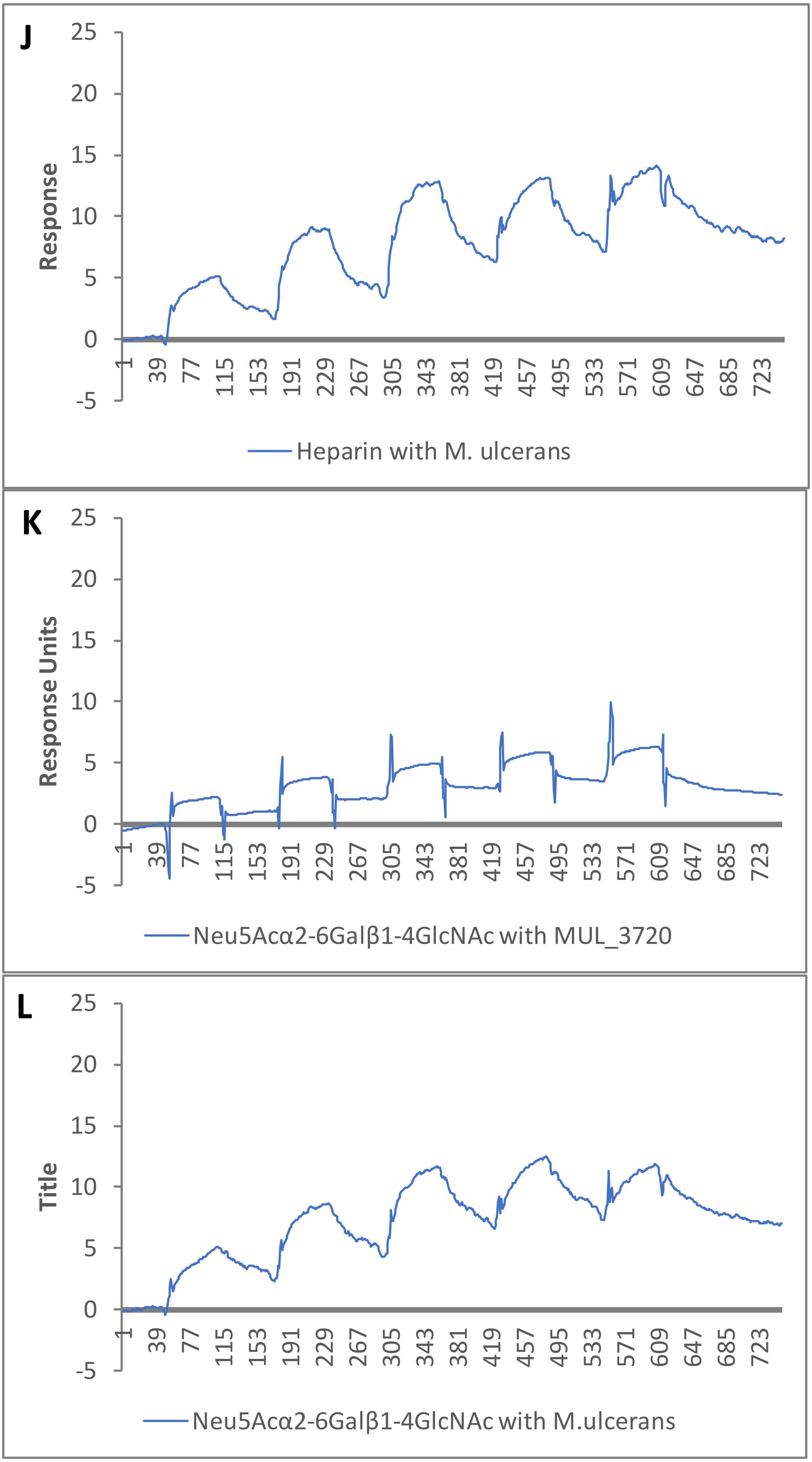
J
Response
25
20
15
10
5
0
-5
1
39
77
115
153
191
229
267
305
343
381
419
457
495
533
571
609
647
685
723
Heparin with M. ulcerans
K
Response Units
25
20
15
10
5
0
-5
1
39
77
115
153
191
229
267
305
343
381
419
457
495
533
571
609
647
685
723
Neu5Acα2-6Galβ1-4GlcNAc with MUL_3720
L
Title
25
20
15
10
5
0
-5
1
39
77
115
153
191
229
267
305
343
381
419
457
495
533
571
609
647
685
723
Neu5Acα2-6Galβ1-4GlcNAc with M.ulcerans

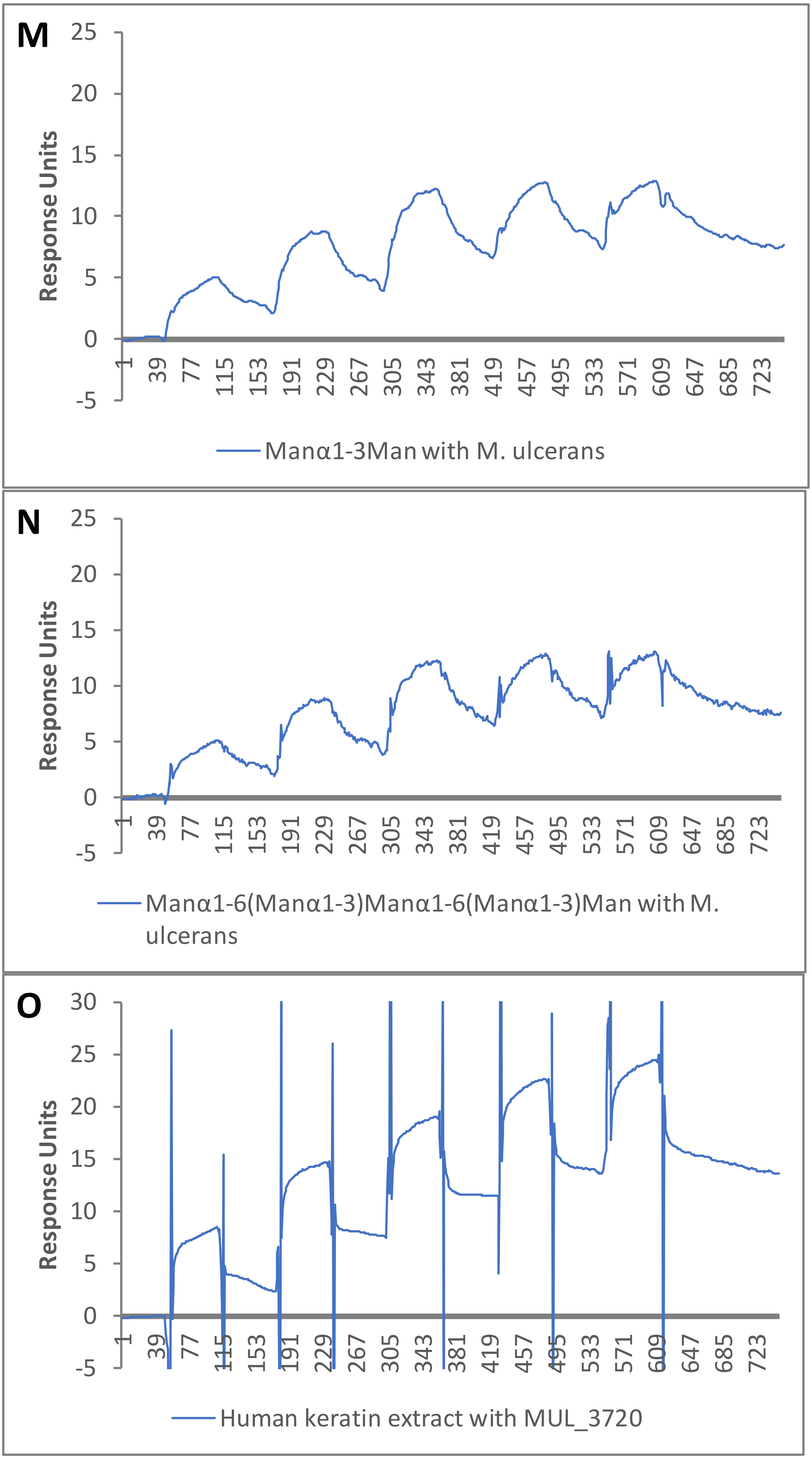
M
Response Units
25
20
15
10
5
0
-5
1
39
77
115
153
191
229
267
305
343
381
419
457
495
533
571
609
647
685
723
Manα1-3Man with M. ulcerans
N
Response Units
25
20
15
10
5
0
-5
1
39
77
115
153
191
229
267
305
343
381
419
457
495
533
571
609
647
685
723
Manα1-6(Manα1-3)Manα1-6(Manα1-3)Man with M. ulcerans
O
Response Units
30
25
20
15
10
5
0
-5
1
39
77
115
153
191
229
267
305
343
381
419
457
495
533
571
609
647
685
723
Human keratin extract with MUL_3720

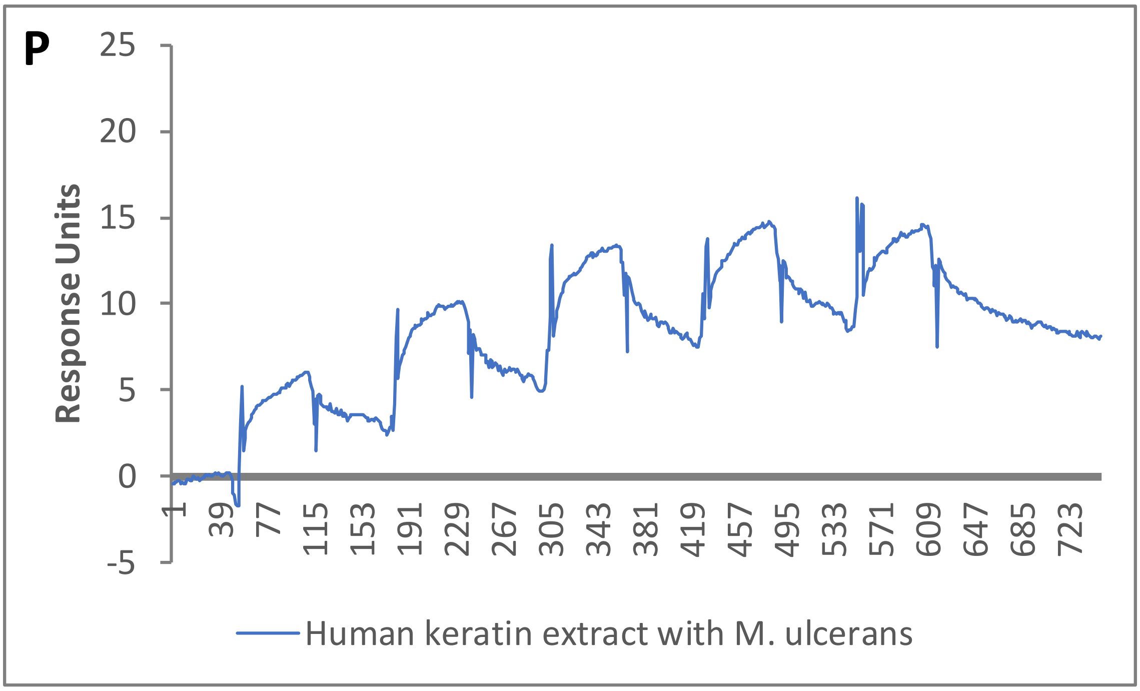

Representative sensor grams of MUL3720 and *M. ulcerans* with glycans and keratin extract. Interactions as indicated on graph with a maximum concentration of: **A**. 20 µM. **B**. 20 µM. **C**. 20 µM. **D**. 20 µM. **E**. 20 µM. **F**. 20 µM. **G**. 1 µM. **H**. 1 µM. **I**. 20 µM. **J**. 20 µM. **K**. 100 nM. **L**. 20 µM. **M**. 1 µM. **N**. 1 µM. **O**. 100 nM. **P**. 100 nM.
