# Supplementary figures and images for: The cell surface protein MUL_3720 confers binding of the skin pathogen Mycobacterium ulcerans to sulfated glycans and keratin
Source: PLoS Negl Trop Dis. 2021 Feb 25;15(2):e0009136. doi: 10.1371/journal.pntd.0009136 (PMC7906334; doi:10.1371/journal.pntd.0009136)

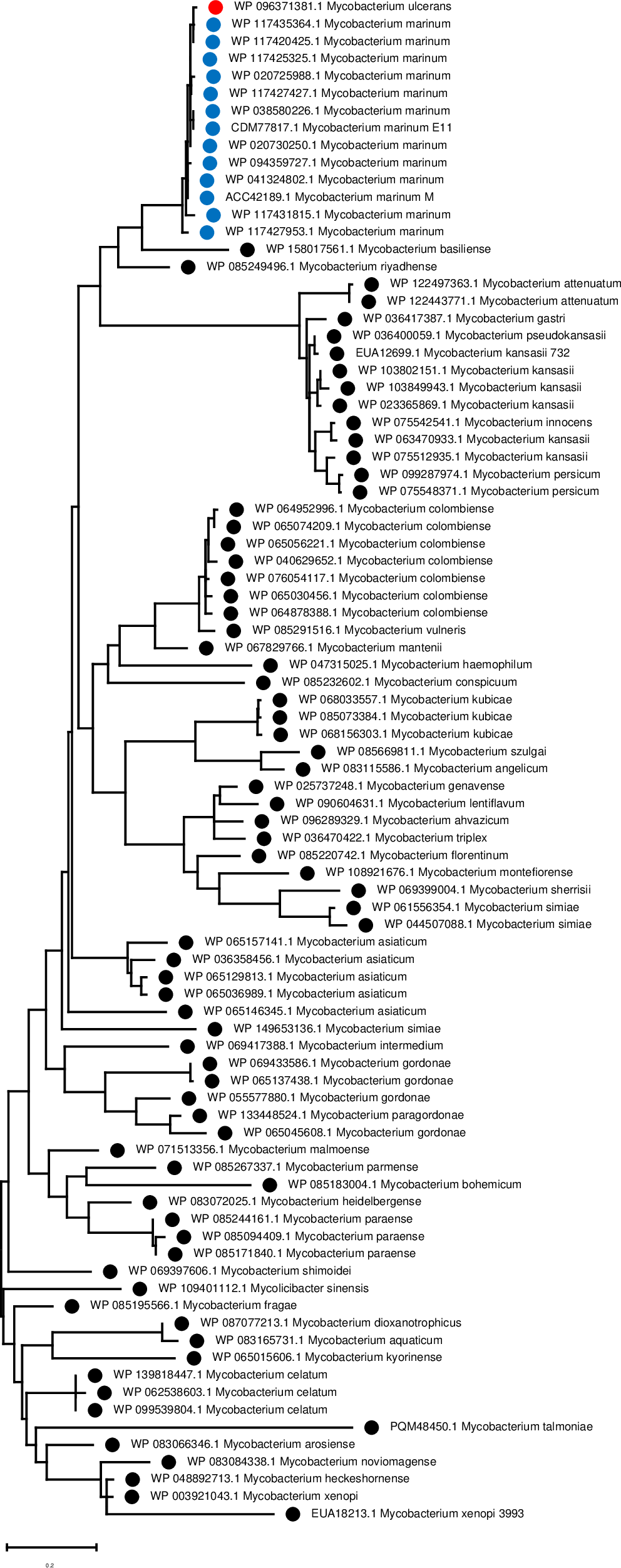

Supplement: S1 Fig — (TIF) [file pntd.0009136.s004.tif]

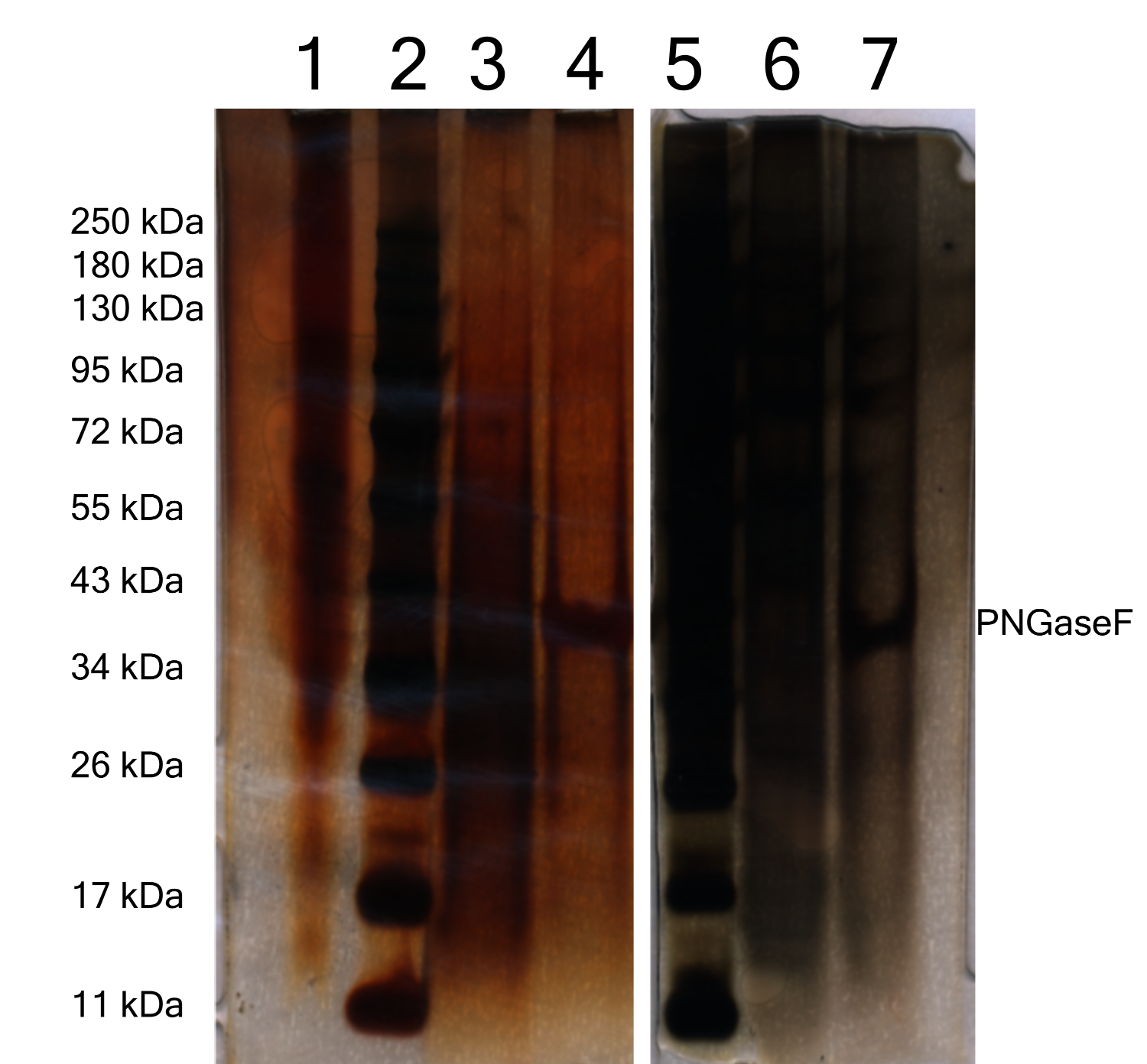

Supplement: S2 Fig — Lane 1 DNA ladder, Lane 2 and 5 NEB prestained blue protein ladder. Lane 3 and 6 Keratin extract without PNGaseF treatment. Lane 4 and 7 Keratin extract with PNGase F treatment. PNGase is labelled on the right side of the gel image. (TIF) [file pntd.0009136.s005.tif]
