# Supplementary material for: The cell surface protein MUL_3720 confers binding of the skin pathogen Mycobacterium ulcerans to sulfated glycans and keratin
Source: PLoS Negl Trop Dis. 2021 Feb 25;15(2):e0009136. doi: 10.1371/journal.pntd.0009136 (PMC7906334; doi:10.1371/journal.pntd.0009136)
Supplement: Attachment — Submitted filename: Response to reviewers final.docx [file pntd.0009136.s006.docx]

Editor Comment: It is essential that the **quantitative data associated with Table 1 should be included in the main body of the manuscript in a format that allows the reader to make quantitative comparisons**. The numbers could be added to the table in addition to actual values for the background spots to assess whether the cutoff is appropriate.

**Table 1 has been updated and the cut offs have been added to the legends of table 1 and Dataset S1.**

Editor Comment: **Additional discussion of the bioinformatics associated with MUL3720 that includes homology to other mycobacterial proteins would be beneficial to bring the reader up to date concerning what is known about this protein beyond the authors’ previous publication.**

**Text has been added to the discussion and a new supplementary figure has also been added to the manuscript. (Page 10; Paragraph 3; line 8)**

**“The MUL_3720 protein was predicted to be a mannose binding lectin [10], however, the glycan array analysis revealed that MUL_3720 is binding to sulphated GAGs and not to mannose structures. MUL_3720 is an orthologue of a predicted lectin found in multiple other mycobacterial species including M. marinum, M . basiliense, M. riyadhense, M. attenuatum, M. gastri, M. pseudokansasii, M. innocens and M. persicum, with the M. marinum orthologue being most similar (99% sequence identity [10]) to the M. ulcerans protein (Fig S1). Like M. ulcerans, M. marinum infections in humans are also typically limited to the skin [41-44], indicating the possiblility that M. marinum MMAR_3773 is targeting similar glycans. M. marinum also causes a tuberculosis like infection in various fish species [41] and sulphated GalNAc structrues such as those found in keratan and chondroitin sulfate are the most common gsulfated GAGs in many fish species [45-47]. The data on MUL_3720 may be indicative for a role of MMAR_3773 in M. marinum infections in both humans and fish. “**

Editor Comment: Additionally the **sensograms produced by the Biacore SPR instrument should be made available as part of this submission for assessment of the kinetics of dissociation as this provides a measure of avidity** which may be equally important for the biological activity of MUL3720 binding.

**Sensorgrams for all data presented in the manuscript are now shown as individual panel within Dataset S2.**

Editor comment: One reviewer was particularly concerned that the **title was inaccurate in the conclusions reached** and that “**Mycobacerium” was misspelled,** so it would be essential to alleviate these concerns.

**We apologise for the spelling error, which has been corrected. The title has been altered to “The cell surface protein MUL_3720 confers binding of the skin pathogen Mycobacterium ulcerans to sulfated glycans and keratin” and the conclusions have also been revised to address reviewer comments as detailed below.**

Reviewer #1: There are some significant issues with the presentation. The **protein expression protocol is a verbatim repetition of an identical paragraph in reference 10.**

**The description of the method has been modified so that is no longer identical (Paragraph 2 of Page 4).**

The **actual synthetic sequence and the primers for PCR are not given either there or here and documentation of the protein purification not shown**.

**We have cited an additional paper that provides details of the primers and the cloning and provides the standard purity of the protein using the method (Paragraph 2 of Page 4).**

Reviewer #3: The authors describe a nice study designed to identify the major glycans interacting with Mycobacterium ulcerans and then test the hypothesis that, based on sequence motifs, MUL_3720 participates in glycan binding. The authors methods for initial identification are straight forward. The authors provide enough information and data for clear understanding of the methods. **The manuscript would benefit from an additional method where binding is competed**; one, due to the conjecture of multiplicity of ligands interacting with the 64 glycans identified for whole cells, and more importantly, to better determine the specific glycans interacting with MUL_3720, and whether there is one, or multiple interaction sites on the protein. This is important because the glycan motif cannot be discerned from the glycans identified alone (there is no consensus motif).

**We have tested the keratin used in the study with a 6S-disaccharide and found that the binding overlaps. This has been added to the results section. (Page 9 line 2)**

**“To determine whether the binding of MUL_3720 to keratin was via the sulfated glycan modification, we conducted competition studies. The chondroitin 6S-disaccharide ΔUAα1-3GalNAc-6S was used as a competimer in SPR binding competition analyses with the keratin extract (Figure 1). These data indicate strongly for an overlap of the binding sites for the 6S-disaccharide and the keratin extract, as the data for the binding to the combined ligands was closer to a combined average than to an additive signal or to one outcompeting the other.”**

Reviewer #2: **The quantitative data associated with Table 1 should be included in the main manuscript**
See additional comments in summary

**Quantitative data has been added to Table 1 as requested by the reviewer.**

Reviewer #3: The authors present data demonstrating that whole M. ulcerans binds to 64 glycans, all of which resemble sulfated glycoconjugates found on epidermal and dermal cells. The authors further present data demonstrating that 7 of these 64 glycans bound to MUL_3720. The data may be a little misleading for a number of these 7 glycans. This is because ~ 1 X 10E6 cells were used versus 2 microgram of recombinant protein. 10E6 mycobacterial cells would equate to roughly 3 micrograms of dehydrated cell biomass, and then at best, 2 micrograms of total protein. Even if MUL_3720 is 10% of the total protein, that would be 0.2 microgram of the total. Thus where the glycan arrays show similar binding and disassociation constants, these are likely not bona fide binders for MUL_3720 (the specific activity would be higher relative to whole cells). The binding comparison to keratin, however, is quite nice and supports their hypothesis.

**The mathematics of the interaction of the amount of protein vs bacteria on the array is not completely accurate as it is not just the total amounts (mass) but also the relative fluorescence of the protein:antibody complex vs the labelled mycobacterium. Not all 64 glycans bound by the bacteria were sulfated, but all that bound by MUL_3720 were. This also assumes attachment of all of the protein/bacteria. This assumption is also false, neither approach binds all or even most of what was added. The exact percentage bound is unknown.**

Reviewer #1: (1) There are significant concerns about the conclusion that the primary target for the mycobacterial lectin is glycosylated keratin. It is indicated that binding it to keratan sulfate oligosaccharides attached to keratin, with reference 15 cited as the basis for claiming the keratin protein is conjugated with glycosaminoglycans. Beyond the work in this 1993 manuscript, there appears to be no other published information on this suggestion and a recent comprehensive review of post-translational modifications of keratins, Nature Reviews Molecular Cell Biology 15, 163–177 (2014), does not mention keratan sulfate. The conclusion in reference 15 is based on immunological analysis, with no biochemical follow-up, and the conclusions is nuanced: "These results demonstrate that a portion of the cytoplasmic anti-keratan sulfate immunoreactivity is due to keratins that are glycosylated with carbohydrates that contain keratan sulfate epitopes or that keratan
sulfate-containing molecules bind or co-migrate in SDS-polyacrylamide gels with cytokeratins."

**We have added additional information and references to this section:**

**In the results (Page 8; 5 lines from bottom of page):**

**“Reports studying keratins isolated from primary human cells rather than cancer cell lines, have reported the presence of keratan sulfate glycans in human keratin extracts [25, 26]. Due to this observation, we analysed the interaction of keratin extracted from human epidermis (mixed keratins with average molecular weight of 64.1 kDa) with MUL_3720 and M. ulcerans cells. The cells bound to the keratin extract with a KD of 37.9 nM (Table 2), on par with interactions observed for the glycans Neu5Acα2-6Galβ1-4GlcNAc and Manα1-3Man. MUL_3720 showed a nearly 10 fold higher affinity to the keratin extract (KD 4.78 nM; Table 2). To determine whether the binding of MUL_3720 to keratin was via the sulfated glycan modification, we conducted competition studies. The chondroitin 6S-disaccharide ΔUAα1-3GalNAc-6S was used as a competimer in SPR binding competition analyses with the keratin extract (Figure 1). These data indicate strongly for an overlap of the binding sites for the 6S-disaccharide and the keratin extract, as the data for the binding to the combined ligands was closer to a combined average than to an additive signal or to one outcompeting the other.”**

**And discussion (Page 11; Paragraph 2):**

**“M. ulcerans infects skin, one of the keratin/keratan-rich tissues of the body, with the epidermal layer being made of keratinocytes and the dermal layer having high keratan sulfate areas around hair follicles and sweat glands/ducts [26, 48]. Furthermore, human skin keratin extracts have indicated that keratin is either strongly associated with or is decorated by keratan sulfate glycosylation [25].**

So even at the time the work was published, the possibility of contamination was highlighted. The lack of any further evidence for such a modification of keratin, as well the difficulty of explaining how a Golgi-specific modification could occur on a cytoplasmic protein, bears out the original caution about the interpretation of these results. The material used in the assays was a commercial preparation of mixed keratins that does not seem to have been further characterized to eliminate the very real possibility of contamination with one or more of the several proteoglycans that bear keratan sulfate chains. It is also not clear how it was kept in solution in the surface plasmon resonance assay, since 8 M urea is required to keep keratins soluble. Given the multiple uncertainties associated with these experimental results and their interpretation, it is premature to conclude that the target for MUL_3720 has been identified.

**We have altered our conclusions, discussions and added a new show that the human skin keratin extract that binds to MUL_3720 in direct competition with sulfated glycans that are known to associate with keratin extracted from human skin. These latter data are demonstrated in a new competition assay (now added to a new Figure 1).**

(2) The presentation of the glycan array results in the main manuscript is without any quantitative information. Examination of the complete results in supplemental information indicates that the dynamic range, from lowest to highest signals, is very limited compared other experiments with this array, such as in reference 16. The actual values are not given for the background spots, making it further difficult to assess whether the cutoff is appropriate.

**All quantitative data has been added to Table 1 and the updated information has been added to Table 1’s legend and the method and MIRAGE-format-compliant supplementary data Table (Table S1).**

(3) There is no comment on a key inconsistency between the array results and the binding competition: extremely high (nM) affinity binding of NeuAc2-6-N-acetyllactosamine to the fixed bacteria, and this ligand has one of the highest (microM) affinities for the expressed protein as reported in Table 2, while this identical oligosaccharide appears as glycan 300 on the array but shows only low background binding in each case. The absence of congruence between two experiments is a concern, particularly since as it stands the results in Table 2 suggest that the common feature of high affinity ligands for MUL_3720 is a negative charge: all the tested ligands with negative charge compete, those without charge do not compete. Additional ligands would need to be tested in this format to make a convincing case for specific binding.

**The reviewer highlights an issue that can be common with analysis between bound and free glycans. Glycan “300” was synthesised with a spacer (spacer is NHCOCH_2_NHCO(CH_2_)_5_NH_2_) then covalent bound to the micro array, wheras glycan “10L” is linked directly to the 12-carbon spacer on the array (the array details are described in reference 18 and are described in the MIRAGE glycan array data deposition table). These spacers are different, and the anomeric fixation of the sugar on the non-reducing end of these arrayed glycans can impact the interaction with test samples . While the non-reducing end sugar is able to adopt multiple different configurations.**

**This difference in the details of glycan coupling chemistry has been addressed in the manuscript:**

**In the revised results section (Page 8; Line 8):**

**“The binding to α2-6 sialyated glycans on the glycan array were confirmed to be differential between M. ulcerans cells and MUL_3720 with the whole bacteria binding to the α2-6Neu5Ac terminating glycan (Neu5Acα2-6Galβ1-4GlcNAc) with ~4600-fold higher affinity than MUL¬3720 (Table 2).”**

**And Discussion (Page 9; Discussion line 6):**

**“The whole M. ulcerans cells were found in SPR analyses to have the highest affinity among the tested glycans for the sialylated Neu5Acα2-6Galβ1-4GlcNAc (Table 2). While this exact glycan was not bound on the array, smaller mono/disaccharide versions of it were tested on the array. Testing using the larger free trisaccharide can reflect the results of smaller sugars. This is due to the flexibility of a glycan that has not been anchored on the non-reducing end, the end normally attached to a protein/lipid/array surface. The non-reducing GlcNAc of this sugar is not in any fixed anomeric configuration (α/β) due to it not being linked. All the glycans on the array have a fixed anomeric configuration and orientation dictated by the linkage to the spacer. This spacer effect has been observed to effect the binding of molecules to glycans printed on arrays previously [27, 28]. It is due to the effect of presentation that testing with a second method such as SPR is so important. In human skin Neu5Acα2-6 is known to be associated with eccrine sweat glands [29], which may be a potential invasion site for M. ulcerans [30]. The high affinity binding of this structure was not due to MUL_3720, and is likely to be mediated by another M. ulcerans lectin.”**

(4) In Table 2, the quantification of affinities based on molar values for polysaccharides such as heparin and chondroitin sulfate is confusing, since these polysaccharides do not have defined molecular weights. The presentation of only "positive" results in Table 1 also masks the fact that the full results in the supplemental table show that other glycans containing similar sulfated residues do not bind. No effort is made to pick apart the differences. The fact that binding is seen to all three different types of glycosaminoglycans, keratan sulfate, chondroitin sulfate and heparin is hard to reconcile with binding to specific sulfate disaccharides.

**All glycans bound by the MUL_3720 protein has a 6-sulfation on either a Gal or GalNAc. As explained in the MIRAGE table, cutoffs used in this assay are likely to produce false negatives from the array analysis, but not false positives. The lack of binding to some 6-sulfated glycans on the array is consisted with our array and subsequent, more sensitive SPR studies.**

(5) It is suggested on page 8 that "glycolipids produced by mycobacterial species may provide another adherence factor that can explain the glycan recognition of M. ulcerans cells in the glycan array experiment" seem to posit binding of glycolipids in the mycobacteria to sugars on the array. Such an interaction would be without precedent.

**We have updated this section and properly referenced glycan-glycan interactions. We apologise for not referencing this section in the previous version of the manuscript as the number of recent references indicates that this kind of interaction is not unprecedented. (Page 10; Paragraph 3).**

**“Mycobacterial species produce numerous cell surface glycan structures with a large proportion of their surface glycosylation being lipid-linked sugars. Recently, glycan-glycan interactions have been identified as a mechanism for pathogen association with host cells [9, 20, 34-37]. Surface glycans such as the lipo-oligo/polysacchride of Gram negative bacteria have been shown to bind to host surface glycans [9, 34, 35, 38, 39]. The extent and diversity of the glycolipids produced by mycobacterial species may thus provide another adherence factor that can explain the glycan recognition of M. ulcerans cells in the glycan array experiment.”**

Reviewer #3: Again, this is a nice paper. It provides quite a nice insight regarding M. ulcerans pathogenesis/infection establishment. The methods need to have some improved rigor, and the conclusions regarding the results need to be provided in the context of the limitations of the comparisons from the glycan arrays. The conclusions should include the need to perform additional glycan studies to identify the bona fide interaction between keratin and the protein.

**We have tested an additional glycan that has greater similarity to those bound on the array. We have also used this disaccharide in competition studies with the keratin extract used in the study and found that the binding overlaps. This has been added to the results section. (Page 9 line 2)**

**“To determine whether the binding of MUL_3720 to keratin was via the sulfated glycan modification, we conducted competition studies. The chondroitin 6S-disaccharide ΔUAα1-3GalNAc-6S was used as a competimer in SPR binding competition analyses with the keratin extract (Figure 1). These data indicate strongly for an overlap of the binding sites for the 6S-disaccharide and the keratin extract, as the data for the binding to the combined ligands was closer to a combined average than to an additive signal or to one outcompeting the other.”**

Reviewer #1: The reference list is also in poor condition, with the names of authors missing in reference 2 and an incomplete citation in reference 19 as well as inconsistent formatting.

**Reference 2 has been fixed, we apologise for this error. The references have been reformatted using Endnote and PLoS.ens. The reformatted references have been checked for correct formatting and as far as we are aware all references are now correct and properly formatted.**

Reviewer #2: The manuscript provides evidence that the M. ulcerans protein MUL3720 binds with keratin, sulfated glycans and glycosaminoglycans. The analyses performed are relatively straight forward and included the binding of M. ulcerans and MUL3720 to glycan arrays, and MUL3720 interactions with a variety of purified glycoproteins and glycans by surface plasmon resonance (SPR). The studies do not, however, demonstrate 1) that MUL3720 is the primary keratin binding ligand of M. ulcerans or that this protein is a factor in the colonization of skin based on keratin binding. Additional concerns with the manuscript are directed at the rationale for the selection of targets and the data presented.

**We have significantly updated our text around the description of the rationale used for picking glycans as outlined above. We have also shown that the sulfated glycan, more like those found on the array and the human keratin extract directly compete for a common site on MUL_3720.**

1) The significance of the work presented if the authors could demonstrate that purified MUL3720 is able to prevent the binding of M. ulcerans to keratin.

**This was attempted, however, there is a strong interaction between MUL_3720 and *M. ulcerans* probably through the LysM domain that makes the competition analysis difficult to obtain. In fact, more binding of keratin the in system was observed when both keratin and MUL_3720 were added. This data has not been added to the manuscript.**

2) The rationale for targeting MUL3720 for glycan binding over other M. ulcerans proteins should be articulated. Is this the only protein of this bacterium with a predicted lectin domain?

**A statement has been added to articulate the lectin binding annotation of MUL_3720 (Page 6; results line 1).**

**“Abhinav et al. (2013) identified 94 lectins in mycobacterial genomes, the only lectin in M. ulcerans determined by this analysis is MUL_3720 [23]. MUL_3720 is annotated as a possible lectin based on the presence of a B-lectin domain that has a mannose binding consensus sequence [10]. Due to this hypothesised lectin activity, both MUL_3720 and whole fixed M. ulcerans cells were analysed for glycan binding activity using a glycan arrays.”**

3) A deeper discussion of the bioinformatics associated with MUL3720 that includes homology to other mycobacterial proteins would be beneficial to the reader.

**This has been added to the discussion. (Page 10; 4 lines from the bottom)**

**“The MUL_3720 protein was predicted to be a mannose binding lectin [10], however, the glycan array analysis revealed that MUL_3720 is binding to sulphated GAGs and not to mannose structures. MUL_3720 is an orthologue of a predicted lectin found in multiple other mycobacterial species including M. marinum, M . basiliense, M. riyadhense, M. attenuatum, M. gastri, M. pseudokansasii, M. innocens and M. persicum, with the M. marinum orthologue being most similar (99% sequence identity [10]) to the M. ulcerans protein (Fig S1). Like M. ulcerans, M. marinum infections in humans are also typically limited to the skin [41-44], indicating the possiblility that M. marinum MMAR_3773 is targeting similar glycans. M. marinum also causes a tuberculosis like infection in various fish species [41] and sulphated GalNAc structrues such as those found in keratan and chondroitin sulfate are the most common gsulfated GAGs in many fish species [45-47]. The data on MUL_3720 may be indicative for a role of MMAR_3773 in M. marinum infections in both humans and fish.”**

4) The glycan Neu5Acα2-6Galβ1-4GlcNAc greater affinity than the sulfated glycan tested. The significance of the interaction with this glycosaminoglycan is not addressed by the authors.

**The binding to Neu5Acα2-6Galβ1-4GlcNAc only has higher affinity when looking at the whole bacteria but is much worse for MUL_3720. The adhesion responsible for the binding and much of the other binding seen for whole *M. ulcerans* is not covered in this manuscript. We have altered the text in the results (Page 7; Paragraph 2):**

**“To validate the glycan array results using a different technique and to determine the dissociation equilibrium constant (KD) of the interactions, SPR analyses were performed between free oligosaccharides and M. ulcerans cells or purified recombinant MUL_3720 that were bound to sensor chips (Table 2). Eight glycan structures representative for each of the distinct glycan groups that either bound only to M. ulcerans cells or to both M. ulcerans cells and purified MUL_3720 were chosen. These structural groups are representatives of blood group antigens (Fucα1-2Gal), Lewis antigens (Fucα1-2Galβ1-4(Fucα1-3)GlcNAc), sulfated glycans (GAGs including chondroitin 6S-complex polymer, chondroitin 62 disaccharide ΔUAα 1-3GalNAc-6S and, heparin and Fucα1-2(3-O-Su)Galβ1-3(Fucα1-4)GlcNAc), mannose structures (Manα1-3Man, Manα1-6(Manα1-3)Manα1-6(Manα1-3)Man) and sialyated glycans (Neu5Acα2-6Galβ1-4GlcNAc). This set of structures provides a broad coverage of the glycan types recognised by both M. ulcerans cells and purified MUL_3720 to aid in the identification of the hierachy of glycan recognition. The SPR analyses reconfirmed that both MUL_3720 and M. ulcerans cells interact with high affinity (KD in the low μM range) with sulfated glycans and GAGs (Table 2).”**

**and Discussion (Page 9; Discussion line 3):**

**“The glycan-binding profile of M. ulcerans demonstrated that this skin pathogen recognises a range of negatively charged glycans common to sulfated GAGs, sialoglycoconjugates and keratans. Much of the binding is to negatively charged modifications (sulfation/sialylation) of carbon-6 of Gal or GalNAc. The whole M. ulcerans cells were found in SPR analyses to have the highest affinity among the tested glycans for the sialylated Neu5Acα2-6Galβ1-4GlcNAc (Table 2). While this exact glycan was not bound on the array, smaller mono/disaccharide versions of it were tested on the array. Testing using the larger free trisaccharide can reflect the results of smaller sugars. This is due to the flexibility of a glycan that has not been anchored on the non-reducing end, the end normally attached to a protein/lipid/array surface. The non-reducing GlcNAc of this sugar is not in any fixed anomeric configuration (α/β) due to it not being linked. All the glycans on the array have a fixed anomeric configuration and orientation dictated by the linkage to the spacer. This spacer effect has been observed to effect the binding of molecules to glycans printed on arrays previously [27, 28]. It is due to the effect of presentation that testing with a second method such as SPR is so important. In human skin Neu5Acα2-6 is known to be associated with eccrine sweat glands [29], which may be a potential invasion site for M. ulcerans [30]. The high affinity binding of this structure was not due to MUL_3720, and is likely to be mediated by another M. ulcerans lectin.”**

5) The Neu5Acα2-6Galβ1-4GlcNAc used in SPR studies is not noted as a glycan tested by glycan array in Table 1. Thus, it is unclear whether this glycan was not included in the array or if it was not found to bind M ulcerans or MUL3720 using the array technology.

**Neu5Acα2-6Galβ-Sp3 was bound on the array as well as several other Neu5Acα2-6 structures. The exact structure Neu5Acα2-6Galβ1-4GlcNAc was just below the cut off for binding indicating that investigation of the Neu5Acα2-6Gal was worthwhile. Text changes to the results section in comment 4 (above) also deal with this response.**

6) The selection of specific glycans to test for MUL3720 binding by SPR does not have a strong rationale.

**We have better outlined the rationale for choosing these glycans. Text changes to the results section in comment 4 (above) also deal with this response.**

7) The discussion specifically mention sulfated glycan structures Array ID# 179, 161, and 151, that were found to bind to MUL3720 using the array technology. These glycans were not tested by SPR.

**Unfortunately, the glycans mentioned are present on the array cannot be obtained commercially or require custom synthesis at great expense. We have however added a disaccharide of chondroitin that contains only a 6S-GalNAc which shows improved affinity for MUL_3720 compared to the other glycans tested. We believe the data provided is sufficient to support the conclusions of the study. (Page 8; line 5)**

**“There is a large difference between the complex 6S-polymer and the 6S-disaccharide of chondroitin, with a greater than 10-fold higher affinity to the 6S-disaccharide for both the M. ulcerans cells and MUL_3720.”**

8) The sensograms produced by the Biacore SPR instrument allows for assessment of the kinetics of dissociation. This provides a measure of avidity which may be equally important for the biological activity of MUL3720 binding. This data should be provided.

**Sensorgrams for all data presented in the manuscript are now shown as individual panel within Dataset S2.**

9) The quantitative data for table 1 should be provided in the main manuscript.

**This data has been added to table 1.**

Reviewer #3: The study was very straightforward, and just needs to be a little more comprehensive prior to publication. Thus the competitive binding assays were suggested; the results of which would provide important data elements regarding the glycan-protein interactions. Finally, since minimally 10 fold excess recombinant protein was used relative to the whole cell assays, the data should be presented in the context of this potential bias.

**We have tested the keratin used in the study with a 6S-disaccharide and found that the binding overlaps. This has been added to the results section. (Page 9 line 2)**

**“To determine whether the binding of MUL_3720 to keratin was via the sulfated glycan modification, we conducted competition studies. The chondroitin 6S-disaccharide ΔUAα1-3GalNAc-6S was used as a competimer in SPR binding competition analyses with the keratin extract (Figure 1). These data indicate strongly for an overlap of the binding sites for the 6S-disaccharide and the keratin extract, as the data for the binding to the combined ligands was closer to a combined average than to an additive signal or to one outcompeting the other.”**
