# Supplementary material for: The cell surface protein MUL_3720 confers binding of the skin pathogen Mycobacterium ulcerans to sulfated glycans and keratin
Source: PLoS Negl Trop Dis. 2021 Feb 25;15(2):e0009136. doi: 10.1371/journal.pntd.0009136 (PMC7906334; doi:10.1371/journal.pntd.0009136)
Supplement: Attachment — Submitted filename: Reviewer responses[65056].docx [file pntd.0009136.s007.docx]

Reviewer #1

**(1) Fundamentally, the response to the previous comments is disappointing. Rather than providing rigorous evidence about the purity of the keratins used in these studies, the title and abstract have been somewhat nuanced about the connection between keratins and keratan sulfate. As it stands, the description "glycans associated with human skin keratin" is vague and could be misleading, as it might just mean glycans that contaminate the keratin preparation.**

We have addressed this with treatment of the human keratin extract with PNGase F. After this treatment, which specifically removes N-glycans, we observe the heterogenous keratin bands between 15 and 192kDa (pre-treatment; average size according to the manufacturer is 63kDa) are reduce molecular weight and exhibit reduced staining. This results is typical of a highly glycosylated heterogenous mixture of glycoproteins.

Original Point 1
**(a) Reference 26 has been added and cited as showing keratan sulfate is attached to keratins. This reference reports a novel form of glycosylation and specifically states "the glycans described here do not resemble keratan sulfate." Citing it in this way misrepresents the conclusion completely.**

We have removed this citation.

**(b) The revision also does not address the point in the original review that it is also not clear how keratins were kept in solution in the surface plasmon resonance assay, given the need for 8 M urea keep them keratins.**

The keratin is diluted directly into the buffer at low concentrations, no keratin precipitates during this procedure as sufficient urea is maintained for the pH required to keep it soluble.

**(a) In Table 2, the quantification of affinities based on molar values for polysaccharides such as heparin and chondroitin sulfate is confusing, since these polysaccharides do not have defined molecular weights. There is no response to this comment.**
We utilise average molecular weight the same as with the keratin. These values have been added to table 2.

**(b) The fact that binding is seen to all three different types of glycosaminoglycans, keratan sulfate, chondroitin sulfate and heparin is hard to reconcile with binding to specific sulfate disaccharides. The response "The lack of binding to some 6-sulfated glycans on the array is consisted [sic] with our array and subsequent, more sensitive SPR studies" is vague. Lowering the cutoff to a level required to detect binding of many glycans containing sulfated galactose and GalNAc, so they are not "false negatives," would mean that roughly 90% of the glycans on the array would be treated as positive for binding.**
The GAGs are present as large molecules with a range of different confirmations, however, the shorter sugars are only able to maintain a limited number of confirmations on the array. Not every 6S structure is likely to present in the correct orientation. This is why the array has multiple versions of each terminal glycan.

**1. In the abstract, "SPR" is stated as an abbreviation. SPR should be spelled out in the abstract.**

This has been corrected.

**2. In the methods, it was hard to find "Keratin isolated from human epidermis (mixed keratins...) from Sigma Adrich. The authors should confirm the description of what was ordered, or provide a catalog #.**

The cat# has been added to the manuscript.

“keratin isolated from human epidermis (mixed keratins with average molecular weight of 64.1 kDa; Sigma-Aldrich, Cat# K0253)”

**3. In the results, in the legend for Table 1, presented is misspelled as "presende"**

This has been fixed with “presende” replaced with presented.

**Reviewer #2: The authors have appropriately addressed my concerns and comments. It is noted that 1) the abbrevriation PNG in the third line of the of the introduction should not be replaced with the full name**

PNG has been replaced with Papua New Guinea in the introduction.

**2) the authors sound be constant in the use of significant figures in Table 2.**

The tables significant figures have been made consistent.
